# Development of a Genomics-Based Approach To Identify Putative Hypervirulent Nontyphoidal Salmonella Isolates: Salmonella enterica Serovar Saintpaul as a Model

**DOI:** 10.1128/msphere.00730-21

**Published:** 2022-01-05

**Authors:** Ruixi Chen, Rachel A. Cheng, Martin Wiedmann, Renato H. Orsi

**Affiliations:** a Department of Food Science, Cornell University, Ithaca, New York, USA; University of California, Davis

**Keywords:** nontyphoidal *Salmonella*, serovar Saintpaul, pathogen detection, SNP clusters, human virulence, comparative genomic analyses, phenotypic characterization, regulatory policy, invasion, intracellular survival

## Abstract

While differences in human virulence have been reported across nontyphoidal *Salmonella* (NTS) serovars and associated subtypes, a rational and scalable approach to identify *Salmonella* subtypes with differential ability to cause human diseases is not available. Here, we used NTS serovar Saintpaul (*S.* Saintpaul) as a model to determine if metadata and associated whole-genome sequence (WGS) data in the NCBI Pathogen Detection (PD) database can be used to identify (i) subtypes with differential likelihoods of causing human diseases and (ii) genes and single nucleotide polymorphisms (SNPs) potentially responsible for such differences. *S.* Saintpaul SNP clusters (*n* = 211) were assigned different epidemiology types (epi-types) based on statistically significant over- or underrepresentation of human clinical isolates, including human associated (HA; *n* = 29), non-human associated (NHA; *n* = 23), and other (*n* = 159). Comparative genomic analyses identified 384 and 619 genes overrepresented among isolates in 5 HA and 4 NHA SNP clusters most significantly associated with the respective isolation source. These genes included 5 HA-associated virulence genes previously reported to be present on Gifsy-1/Gifsy-2 prophages. Additionally, premature stop codons in 3 and 7 genes were overrepresented among the selected HA and NHA SNP clusters, respectively. Tissue culture experiments with strains representing 4 HA and 3 NHA SNP clusters did not reveal evidence for enhanced invasion or intracellular survival for HA strains. However, the presence of *sodCI* (encoding a superoxide dismutase), found in 4 HA and 1 NHA SNP clusters, was positively correlated with intracellular survival in macrophage-like cells. *Post hoc* analyses also suggested a possible difference in intracellular survival among *S.* Saintpaul lineages.

**IMPORTANCE** Not all *Salmonella* isolates are equally likely to cause human disease, and *Salmonella* control strategies may unintentionally focus on serovars and subtypes with high prevalence in source populations but are rarely associated with human clinical illness. We describe a framework leveraging WGS data in the NCBI PD database to identify *Salmonella* subtypes over- and underrepresented among human clinical cases. While we identified genomic signatures associated with HA/NHA SNP clusters, tissue culture experiments failed to identify consistent phenotypic characteristics indicative of enhanced human virulence of HA strains. Our findings illustrate the challenges of defining hypo- and hypervirulent *S.* Saintpaul and potential limitations of phenotypic assays when evaluating human virulence, for which *in vivo* experiments are essential. Identification of *sodCI*, an HA-associated virulence gene associated with enhanced intracellular survival, however, illustrates the potential of the framework and is consistent with prior work identifying specific genomic features responsible for enhanced or reduced virulence of nontyphoidal *Salmonella*.

## INTRODUCTION

Salmonella enterica causes the greatest estimated burden of all foodborne diseases worldwide (1). While the genus *Salmonella* includes just two species (*enterica* and *bongori*), an appreciable diversity of serological variants, called serovars, make *Salmonella* one of the most diverse foodborne pathogens, with >2,600 recognized serovars (2). Two serovars have been used as models for the two general clinical presentations of salmonellosis in humans. Infection with Salmonella enterica subsp. *enterica* serovar (abbreviated “*S.*”) Typhi, the model for typhoidal salmonellosis or typhoid fever, is associated with an invasive infection and an estimated case mortality rate of 1% (3). Infection with nontyphoidal *Salmonella* (NTS) serovars (i.e., all serovars except for Typhi and Paratyphi A, B, or C), such as the model *S.* Typhimurium, is associated with a self-limiting gastroenteritis, with a considerably lower rate of case fatality (estimated case fatality, 0.0003 to 0.003%) (4, 5). Nontyphoidal serovars are often able to infect a broad range of hosts (6) and, as such, present a challenge to control efforts.

Not all *Salmonella* serovars are equally likely to cause human clinical disease. For example, some serovars such as *S.* Cerro are commonly isolated from agricultural animals (7) but are rarely associated with human clinical cases (8). Furthermore, some NTS serovars are associated with considerably higher rates of invasive disease (9), suggesting that controlling these serovars will likely have a bigger impact on improving public health outcomes. Due in part to the current regulatory approaches that typically consider all Salmonella serovars uniformly, without accounting for potential differences in likelihood of causing human clinical disease, control strategies may have unintentionally focused on the control of serovars that are prevalent in a common agricultural commodity but are not necessarily likely to cause human clinical disease (10). Combined with a lack of progress on reducing the overall incidence of human salmonellosis in the United States (11), this highlights the opportunity for revised approaches to more effectively reduce the public heath impact of Salmonella in the food supply by focusing control efforts on those subtypes that are more likely to cause human disease and not expending resources on control of subtypes that are unlikely to cause human disease.

We recently proposed a framework for using whole-genome sequence data to identify genomic signatures that are over- or underrepresented among isolates assigned to NCBI Pathogen Detection (PD) single nucleotide polymorphism (SNP) clusters with high and low proportions of human clinical isolates, combined with phenotypic experiments to (i) identify phenotypic characteristics reflective of the association of the SNP clusters with human clinical cases and (ii) understand contributions of the identified genomic signatures to pathogenesis, as a modernized approach to controlling Salmonella based on likelihood of causing human clinical disease (10). To demonstrate the implementation of such an approach, we selected *S.* Saintpaul as a model, because (i) this serovar is understudied despite the fact that its incidence among reported human clinical salmonellosis cases has increased from 577 reported cases in 2006 to 778 in 2016 (8), (ii) it has been associated with several large outbreaks, including one linked to fresh produce in 2008 that sickened 1,500 people (12), and (iii) despite the observation that NCBI PD SNP clusters within this serovar vary drastically in the proportion of human clinical isolates, there is limited information on virulence differences among *S.* Saintpaul subtypes. Using data for NCBI PD SNP clusters, we classified *S.* Saintpaul SNP clusters into three epidemiology types (epi-types): (i) human-associated (HA) SNP clusters, which have significantly higher proportions of human clinical isolates, (ii) non-human-associated (NHA) SNP clusters, which have significantly lower proportions of human clinical isolates, and (iii) other SNP clusters, which did not show significant over- or underrepresentation of human clinical isolates. We then used comparative genomic analyses to identify genes and core SNPs overrepresented among SNP clusters representing HA or NHA epi-types and used a selected set of strains to phenotypically assess whether these epi-types or genomic signatures found to be overrepresented in these epi-types were associated with the ability of *S.* Saintpaul to invade human intestinal epithelial cells and/or to survive inside human macrophage-like cells.

## RESULTS

### *S.* Saintpaul includes SNP clusters with significant over- or underrepresentation of human clinical isolates.

We used whole-genome sequencing (WGS)-associated metadata available on the NCBI PD platform to calculate odds ratios and associated adjusted *P* values for *S.* Saintpaul SNP clusters having differential proportions of human clinical isolates. A total of 4,759 *S.* Saintpaul isolates (accessed 23 June 2020), which were assigned to 211 SNP clusters, were available in the database. Human clinical isolates were significantly (Benjamini-Hochberg [BH]-corrected *P < *0.05) overrepresented among isolates assigned to 29 SNP clusters (designated HA SNP clusters) and were significantly underrepresented among isolates assigned to 23 SNP clusters (designated NHA SNP clusters) (Table 1; see also Data Set S1, tab 1, in the supplemental material for the full list of *S.* Saintpaul SNP clusters), indicating that *S.* Saintpaul isolates assigned to different SNP clusters may vary in their likelihood for causing human illness (i.e., clinical disease).

**TABLE 1:** *S.* Saintpaul SNP clusters with significantly higher or lower proportions of human clinical isolates

SNP cluster	Epi-type^*a*^	No. of isolates			Odds ratio^*b*^	*P* value^*c*^
		Human	Nonhuman	Total		
PDS000006321.200^*d*^	HA	388	13	401	12.933	<0.001
PDS000029281.36	HA	241	0	241	Inf	<0.001
PDS000004371.140^*d*^	HA	203	6	209	13.885	<0.001
PDS000009466.139^*d*^	HA	213	14	227	6.225	<0.001
PDS000031607.43	HA	108	0	108	Inf	<0.001
PDS000002536.90^*d*^	HA	121	3	124	16.175	<0.001
PDS000006544.8	HA	71	0	71	Inf	<0.001
PDS000032614.60^*d*^	HA	115	10	125	4.579	<0.001
PDS000003801.90	HA	117	13	130	3.577	<0.001
PDS000009725.44	HA	50	1	51	19.656	<0.001
PDS000002279.52	HA	66	4	70	6.503	<0.001
PDS000013987.46	HA	51	2	53	10.020	<0.001
PDS000028639.6	HA	44	1	45	17.266	<0.001
PDS000029315.14	HA	69	6	75	4.529	<0.001
PDS000022946.21	HA	33	0	33	Inf	<0.001
PDS000004376.21	HA	31	0	31	Inf	<0.001
PDS000029174.10	HA	30	0	30	Inf	<0.001
PDS000028163.22	HA	37	1	38	14.489	<0.001
PDS000029326.49	HA	96	14	110	2.706	0.001
PDS000028460.3	HA	25	0	25	Inf	0.002
PDS000042733.19	HA	23	0	23	Inf	0.003
PDS000029489.19	HA	22	0	22	Inf	0.004
PDS000001869.65	HA	73	11	84	2.607	0.005
PDS000002283.9	HA	21	0	21	Inf	0.005
PDS000014879.19	HA	21	0	21	Inf	0.005
PDS000003803.30	HA	29	2	31	5.660	0.015
PDS000030636.11	HA	17	0	17	Inf	0.018
PDS000028687.19	HA	22	1	23	8.577	0.024
PDS000029061.13	HA	16	0	16	Inf	0.024
PDS000004383.106^*d*^	NHA	2	366	368	0.002	<0.001
PDS000029303.44^*d*^	NHA	70	164	234	0.148	<0.001
PDS000004385.43^*d*^	NHA	9	90	99	0.036	<0.001
PDS000032619.10^*d*^	NHA	1	72	73	0.005	<0.001
PDS000004163.70	NHA	20	63	83	0.118	<0.001
PDS000001868.151	NHA	140	146	286	0.345	<0.001
PDS000003800.23	NHA	14	43	57	0.123	<0.001
PDS000030862.1	NHA	1	25	26	0.015	<0.001
PDS000032172.2	NHA	2	13	15	0.059	<0.001
PDS000023865.11	NHA	10	17	27	0.226	0.001
PDS000037793.7	NHA	10	17	27	0.226	0.001
PDS000011234.6	NHA	0	6	6	0.000	0.003
PDS000037718.2	NHA	0	6	6	0.000	0.003
PDS000032576.18	NHA	18	21	39	0.329	0.003
PDS000002534.6	NHA	1	7	8	0.055	0.005
PDS000025969.3	NHA	0	5	5	0.000	0.009
PDS000041109.2	NHA	2	7	9	0.110	0.014
PDS000002533.6	NHA	0	4	4	0.000	0.025
PDS000004372.6	NHA	0	4	4	0.000	0.025
PDS000030850.1	NHA	0	4	4	0.000	0.025
PDS000032145.1	NHA	0	4	4	0.000	0.025
PDS000048106.3	NHA	0	4	4	0.000	0.025
PDS000037705.8	NHA	2	6	8	0.129	0.031

a Epi-type is assigned based on the significant over- or underrepresentation of human clinical isolates among all isolates assigned to a given SNP clusters. For HA, the SNP clusters show a significant overrepresentation of human clinical isolates; for NHA, the SNP clusters show a significant underrepresentation of human clinical isolates. Inf, infinity.

b The odds ratio of a given SNP cluster refers to the ratio of the odds of human clinical isolates in the SNP cluster to the odds of human clinical isolates not in the SNP cluster.

c BH-corrected *P* value of the Fisher’s exact tests.

d SNP clusters that are included in the comparative genomic analyses in this study.

10.1128/mSphere.00730-21.5 DATA SET S1 Statistics of *S.* Saintpaul SNP clusters and the 100 SNP clusters of Salmonella enterica subsp. *enterica* serovars that have the greatest number of isolates in the NCBI PD database (accessed 20 February 2020). Tab 1 shows the statistics used to assess the association between each *S.* Saintpaul SNP cluster and human clinical salmonellosis cases, as well as the final epi-type assigned; tab 2 shows the proportion of human clinical isolates of each of the top 100 SNP clusters, as well as the associated statistics. Download Data Set S1, XLSX file, 0.05 MB. Copyright © 2022 Chen et al. 2022 Chen et al. https://creativecommons.org/licenses/by/4.0/ This content is distributed under the terms of the Creative Commons Attribution 4.0 International license.

### Phylogenetic analyses suggest that *S.* Saintpaul is polyphyletic, although the majority of SNP clusters and isolates are assigned to a single phylogenetic group.

**FIG 1:**
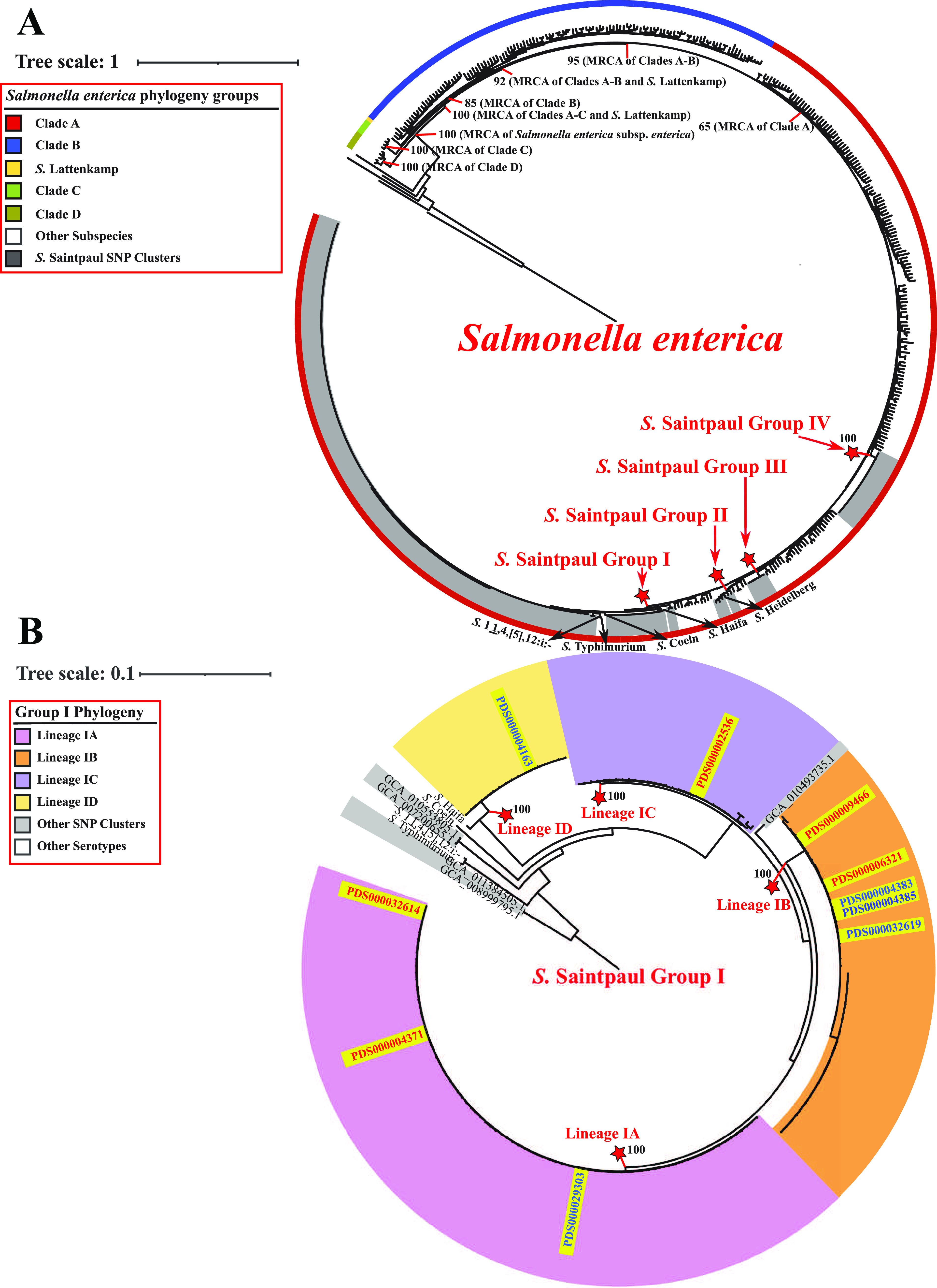
Phylogenetic analyses reveal a polyphyletic structure for *S.* Saintpaul. (A) Maximum likelihood phylogenetic tree inferred from core SNPs among representative isolates for 211 *S*. Saintpaul SNP clusters and reference isolates for (i) 313 unique Salmonella enterica subsp. *enterica* serovars and (ii) 5 additional Salmonella enterica subspecies. Clustering confidence was assessed using 1,000 bootstrap repetitions. The tree is rooted using the reference isolate (assembly accession no. GCA_000018625) of Salmonella enterica subsp. *arizonae* as the outgroup. Branch lengths represent the average pairwise number of nucleotide substitutions per site. Internal nodes indicating the most recent common ancestors (MRCAs) of Salmonella enterica subsp. *enterica* clades are labeled with the corresponding bootstrap values. All *S.* Saintpaul SNP clusters fall within clade A of Salmonella enterica subsp. *enterica* and belong to one of four phylogenetic groups (designated *S.* Saintpaul groups I to IV; gray sections). The ancestral node of each *S.* Saintpaul phylogenetic group is marked by a red star, and the exact bootstrap value of the node is shown. (B) Maximum likelihood phylogenetic tree constructed based on core SNPs for all representative isolates and reference isolates under the *S.* Saintpaul group I ancestral node shown in panel A. Clustering confidence was assessed using 1,000 bootstrap repetitions. The tree is rooted using two *S.* Saintpaul representative isolates (assembly accession no. GCA_011384505.1 and GCA_008999795.1) as outgroups. Branch lengths represent the average pairwise number of nucleotide substitutions per site. Most *S*. Saintpaul SNP clusters within group I belong to one of the four monophyletic lineages (designated lineages IA to ID). The ancestral node of each group I lineage is marked by a red star, and the exact bootstrap value of the node is shown. The 5 HA and 5 NHA SNP clusters that had the most significant association with the corresponding isolation sources are all positioned within group I and marked in red and blue at the corresponding tree leaves, respectively.

To assess the phylogeny of *S.* Saintpaul, we inferred a maximum likelihood phylogenetic tree from the alignment of core SNPs for representative isolates for the 211 *S.* Saintpaul SNP clusters, 313 reference isolates representing unique Salmonella enterica subsp. *enterica* serovars, and 5 additional reference isolates representing the other recognized Salmonella enterica subspecies (Fig. 1A; see Data Set S2, tab 1, for the full list of the representative isolates and reference isolates). Although the genomes of 17 *S*. Saintpaul representative isolates were not sequenced using the Illumina instrument and/or not assembled using the program SKESA, no distinct pattern was observed for these isolates, as they clustered intermixed with the other isolates (data not shown). The tree topology suggested that *S.* Saintpaul is a polyphyletic serovar within S. enterica subsp. *enterica* clade A (13, 14) comprising 4 distinct phylogenetic groups (designated groups I to IV based on tree topology and bootstrap support values) (Fig. 1A). *S.* Saintpaul groups III and IV are monophyletic, containing 6 SNP clusters (total number of isolates, 20) and 22 SNP clusters (total number of isolates, 814), respectively; *S.* Saintpaul group II represents a paraphyletic group containing 5 SNP clusters (total number of isolates, 10) as well as the representative isolate for *S.* Heidelberg; *S.* Saintpaul group I represents a paraphyletic group containing 178 SNP clusters (total number of isolates, 3,867) as well as the representative isolates for *S.* Haifa, *S.* Coeln, *S.* Typhimurium, and *S.* I 1,4,[5],12:i:- (Table S1).

10.1128/mSphere.00730-21.1 TABLE S1 Summary of the number of isolates and SNP clusters by phylogenetic groups and lineages. Download Table S1, DOCX file, 0.02 MB. Copyright © 2022 Chen et al. 2022 Chen et al. https://creativecommons.org/licenses/by/4.0/ This content is distributed under the terms of the Creative Commons Attribution 4.0 International license.

10.1128/mSphere.00730-21.6 DATA SET S2 Statistics of *S.* Saintpaul representative isolates and reference isolates of other serovars. Tab 1 shows the information for isolates included in the phylogenetic analyses, including one representative isolate of each *S.* Saintpaul SNP cluster and one reference isolate of each of the other 318 serovars; tab 2 shows the information for isolates (*n* = 10) selected from each of the 5 HA and 5 NHA SNP clusters with the most significant association with their corresponding isolation sources. Download Data Set S2, XLSX file, 0.1 MB. Copyright © 2022 Chen et al. 2022 Chen et al. https://creativecommons.org/licenses/by/4.0/ This content is distributed under the terms of the Creative Commons Attribution 4.0 International license.

The majority of the SNP clusters that showed over- or underrepresentation of human clinical isolates were classified into group I (24 HA and 21 NHA SNP clusters compared to 5 HA and 2 NHA SNP clusters in group IV) (Table S1). Importantly, 5 HA and 5 NHA SNP clusters within group I showed the most significant association (i.e., had the smallest Fisher’s exact test *P* values) with the corresponding isolation sources among all *S.* Saintpaul SNP clusters, excluding clusters that were likely predominantly represented by isolates from a single outbreak (Table 1). The odds ratios of human clinical isolates among these group I SNP clusters ranged from 4.579 to 16.175 for the HA SNP clusters and from 0.002 to 0.148 for the NHA SNP clusters (Table 1). Together, these observations suggested that further comparative genomic analyses for identification of genomic signatures and phenotypic characteristics associated with the human hypo- and hypervirulence of *S.* Saintpaul should be focused primarily on group I.

To determine the appropriateness for selecting the 5 HA and 5 NHA SNP clusters showing the most significant association with the corresponding isolation sources for use in the comparative genomic analyses, we further assessed the phylogeny of the *S.* Saintpaul group I isolates. The majority of *S.* Saintpaul SNP clusters within group I can be grouped into 4 monophyletic lineages (lineages IA to ID). Lineage IA consists of 80 SNP clusters (total number of isolates, 1,708), lineage IB consists of 46 SNP clusters (total number of isolates, 1,408), lineage IC consists of 30 SNP clusters (total number of isolates, 384), and lineage ID consists of 17 SNP clusters (total number of isolates, 310), which are clustered with *S.* Haifa (bootstrap value, 100) (Fig. 1B). The 5 HA and 5 NHA SNP clusters are distributed among all four lineages (Fig. 1B), suggesting a diverse genetic representation of the major phylogenetic lineages of *S.* Saintpaul group I. Notably, one NHA SNP cluster (PDS000004163) belongs to lineage ID, indicating that isolates within this SNP cluster are more closely related to serovar Haifa than to the other selected HA and NHA SNP clusters. Indeed, the average core SNP distance between the representative isolates in HA and NHA SNP clusters belonging to lineages IA to IC was considerably lower (1,745 core SNP differences) than the average core SNP distance between these lineage IA to IC representative isolates and those from the NHA SNP cluster PDS000004163 (12,332 core SNP differences) (Fig. S1). Due to the high level of genetic dissimilarity between isolates assigned to PDS000004163 and those assigned to the other 5 HA and 4 NHA SNP clusters, this specific SNP cluster was excluded from further comparative genomic analyses and phenotypic characterizations, which were consequently performed using representative isolates from the 5 HA and 4 NHA SNP clusters from lineages IA to IC that showed the smallest *P* values for differential association.

10.1128/mSphere.00730-21.3 FIG S1 Heatmap display of the core SNP distances among the representative isolates (*n* = 10 per SNP cluster) from the 5 HA (marked in red) and 5 NHA (marked in blue) SNP clusters that have the most significant association with the corresponding isolation sources, as well as the reference isolates for *S.* Coeln and *S.* Haifa. SNP clusters and the corresponding lineages are shown at the top of the heatmap (red, HA SNP clusters; blue, NHA SNP clusters). Representative isolates of the NHA SNP cluster PDS000004163 (lower left corner) show larger core SNP distances to the other 5 HA and 4 NHA SNP clusters, and therefore this SNP cluster was excluded from the downstream genomic and phenotypic characterizations. Download FIG S1, PDF file, 1.0 MB. Copyright © 2022 Chen et al. 2022 Chen et al. https://creativecommons.org/licenses/by/4.0/ This content is distributed under the terms of the Creative Commons Attribution 4.0 International license.

### Virulence genes carried on Gifsy-1 and Gifsy-2 prophages are overrepresented among *S.* Saintpaul isolates in HA SNP clusters.

Next, we used comparative genomic analyses to determine if the presence or absence of specific genes may be responsible for the over- or underrepresentation of human clinical isolates in the 5 HA and 4 NHA SNP clusters with the smallest *P* values for differential association. To account for potential biases due to differences in the number of isolates in each SNP cluster, we selected 10 representative isolates from each SNP cluster for comparisons (see Data Set S2, tab 2, for the full list of representative isolates). The pangenome of the 90 representative isolates from the 5 HA and 4 NHA SNP clusters comprised 6,485 genes (see Data Set S3, tab 1, for the full list of gene families in the pangenome), including 3,988 core genes (i.e., genes present in 100% of the isolates) and 2,497 accessory genes (i.e., genes present in <100% of the isolates), which can be further divided into 2,104 shell genes (i.e., genes present in 15 to 99% of the isolates) and 393 cloud genes (i.e., genes present in <15% of the isolates) (Fig. S2). Roughly 40% of the accessory genes (1,003 genes) were associated with isolates representing HA or NHA SNP clusters (designated HA and NHA isolates, respectively), with 384 and 619 genes significantly (BH-corrected *P < *0.05) overrepresented among isolates in HA SNP clusters (designated “HA genes”) and NHA SNP clusters (designated “NHA genes”), respectively (Data Set S3, tab 2).

10.1128/mSphere.00730-21.4 FIG S2 Assessment plots for the pangenome estimation among all 90 *S.* Saintpaul representative isolates of the 5 HA and 4 NHA SNP clusters included in the comparative genomic analyses. (A) Composition of the pangenome. Core genes are present in 100% of the isolates, shell genes are present in 15 to 99% of the isolates, and cloud genes are present in <15% of the isolates. (B) Rarefaction curves showing the number of pan (purple) and core (green) genes with the addition of each successive genome (90 genomes in total). (C) Heatmap showing the presence/absence pattern across the representative isolates for each gene in the pangenome. Download FIG S2, PDF file, 0.5 MB. Copyright © 2022 Chen et al. 2022 Chen et al. https://creativecommons.org/licenses/by/4.0/ This content is distributed under the terms of the Creative Commons Attribution 4.0 International license.

10.1128/mSphere.00730-21.7 DATA SET S3 Statistics of gene families identified among the 90 isolates included in the comparative genomic analysis. Tab 1 shows the information for each gene family in the pangenome estimated among the 90 isolates; tab 2 shows additional information for the gene families significantly over- or underrepresented among HA/NHA isolates. Download Data Set S3, XLSX file, 5.0 MB. Copyright © 2022 Chen et al. 2022 Chen et al. https://creativecommons.org/licenses/by/4.0/ This content is distributed under the terms of the Creative Commons Attribution 4.0 International license.

**FIG 2:**
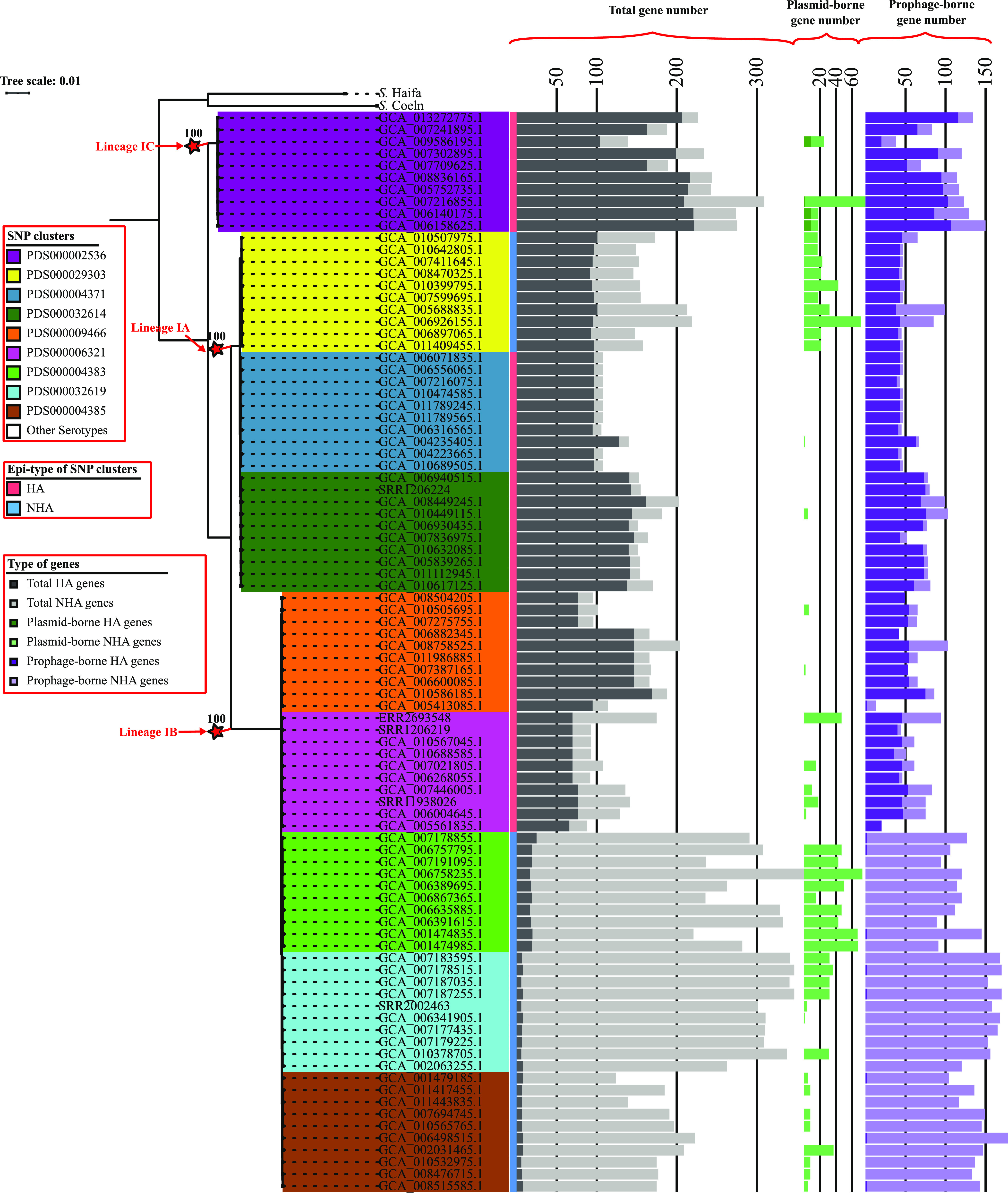
Genes that are overrepresented among HA or NHA isolates may be attributed to plasmids and prophages. Maximum likelihood phylogeny is inferred from core SNPs among 90 *S.* Saintpaul isolates representing 5 HA and 4 NHA SNP clusters, respectively. Clustering confidence was assessed using 1,000 bootstrap repetitions. The tree is rooted using the reference isolate of *S.* Haifa (assembly accession no. GCA_006378355) and *S.* Coeln (assembly accession no. GCA_008488945) as outgroups. Branch lengths represent the average pairwise number of nucleotide substitutions per site. The ancestral node of each lineage is marked by a red star, and the exact bootstrap value of the node is shown. Bar graphs to the right of the phylogeny show, for each isolate, the number of total genes (dark gray, HA; light gray, NHA), plasmid-borne genes (dark green, HA; light green, NHA), and prophage-borne genes (dark purple, HA; light purple, NHA) present in the genomes that are overrepresented among HA or NHA isolates.

Mobile genetic elements (MGEs) represent important components of the accessory genome (15). As plasmids and prophages, two common types of MGEs, have been reported to facilitate the adaptation of Salmonella to human- and non-human-associated environments (16, 17), we assessed their contribution to the association of HA and NHA SNP clusters with their respective isolation sources by identifying and enumerating the number of plasmid-borne and prophage-borne HA/NHA genes for each isolate. The median number of NHA genes per NHA SNP cluster (across the 10 representative isolates selected for the given SNP cluster) ranged from 59 (SNP cluster PDS000029303) to 318 (PDS000032619) (Fig. 2 and Table S2); among these genes, 4.6% (PDS000004385) to 35.6% (PDS000029303) were associated with plasmids (see Data Set S4, tab 1, for the full list of contigs classified as plasmid sequences for each isolate, together with the associated HA/NHA genes), and 19.1% (PDS000029303) to 79.9% (PDS000004385) were associated with prophages (see Data Set S4, tab 2, for the full list of prophage regions identified for each isolate, together with the associated HA/NHA genes). Of note, the number of NHA genes was considerably lower for one NHA SNP cluster, PDS000029303 (median number of NHA genes, 59) than for the other NHA SNP clusters (median numbers of NHA genes, 265, 174, and 318 for PDS000004383, PDS000004385, and PDS000032619, respectively); isolates representing PDS000029303 also possessed a considerably higher proportion of plasmid-borne NHA genes and a considerably lower proportion of prophage-borne NHA genes than that of isolates representing other NHA SNP clusters. These differences may suggest that different mechanisms are responsible for the association of NHA SNP clusters with nonhuman environments. The median number of HA genes per HA SNP cluster ranged from 70 (PDS000006321) to 208 (PDS000002536) (Fig. 2 and Table S2). Among these genes, the proportion of the HA genes carried on prophages ranged from 36.4% (PDS000009466) to 65.7% (PDS000006321), while plasmid-borne HA genes were identified in only 4 isolates from SNP cluster PDS0000025364. These results indicate that prophage-borne genes may contribute to the association of HA and NHA isolates with their corresponding isolation sources, while plasmid-borne genes are more likely to be involved in the ability of the NHA isolates to survive in nonhuman environments.

10.1128/mSphere.00730-21.2 TABLE S2 Range and median number of HA/NHA-associated genes across the representative isolates of each SNP cluster. Download Table S2, DOCX file, 0.02 MB. Copyright © 2022 Chen et al. 2022 Chen et al. https://creativecommons.org/licenses/by/4.0/ This content is distributed under the terms of the Creative Commons Attribution 4.0 International license.

10.1128/mSphere.00730-21.8 DATA SET S4 Statistics of plasmid-borne and prophage-borne sequences. Tab 1 shows the information for contigs of each isolate that were classified as plasmid sequences; tab 2 shows the information for prophage regions identified in the genome of each isolate. Download Data Set S4, XLSX file, 0.2 MB. Copyright © 2022 Chen et al. 2022 Chen et al. https://creativecommons.org/licenses/by/4.0/ This content is distributed under the terms of the Creative Commons Attribution 4.0 International license.

**FIG 3:**
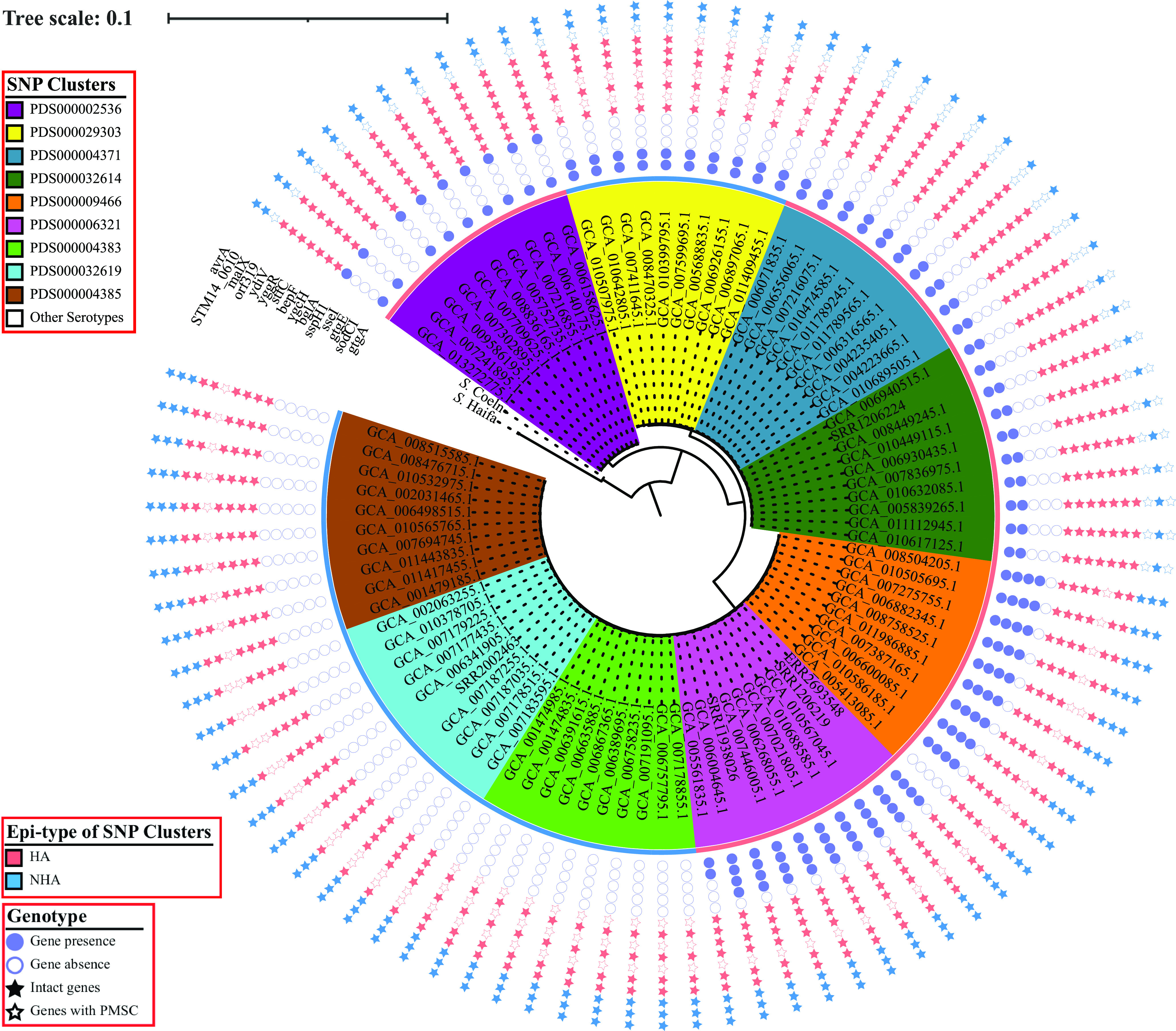
Selected genomic signatures may be responsible for the association of HA or NHA SNP clusters with their corresponding isolation sources. The inner section shows the maximum likelihood phylogeny inferred from core SNPs among 90 *S.* Saintpaul isolates representing 5 and 4 HA and NHA SNP clusters, respectively. Clustering confidence is assessed using 1,000 bootstrap repetitions. The tree is rooted using the reference isolates of *S.* Haifa (assembly accession no. GCA_006378355) and *S.* Coeln (assembly accession no. GCA_008488945) as outgroups. Branch lengths represent the average pairwise number of nucleotide substitutions per site. The ancestral node of each lineage is marked by a red star, and the exact bootstrap value of the node is shown. The outer section shows the genotypic patterns across the isolates with respect to the selected genomic signatures; these include (i) virulence-associated genes carried on prophages Gifsy-1/Gifsy-2 (denoted by circles), (ii) core-SNP-affected genes that tend to be intact in HA isolates while disrupted by a PMSC in NHA isolates (denoted by red stars), and (iii) core-SNP-affected genes that tend to be intact in NHA isolates while disrupted by a PMSC in HA isolates (denoted by blue stars).

When the annotation of the HA genes was manually inspected, we identified 5 HA genes that are carried on lambda prophages Gifsy-1 and Gifsy-2 and have been previously associated with virulence, including *gtgA*, *sodCI*, *sseI*, *gtgE*, and *sspH1* (Fig. 3 and Table 2). This indicates the potential of Gifsy-1 and Gifsy-2 prophages to be associated with the observed overrepresentation of human clinical isolates in HA SNP clusters.

**TABLE 2:** Prophage-borne virulence genes overrepresented among isolates from HA SNP clusters

Gene	Prophage	Odds ratio^*a*^	*P* value^*b*^	Gene product and associated functions	Reference(s)
*sodCI*	Gifsy-2	10.64	<0.001	Periplasmic Cu-Zn superoxide dismutase; reacts with phagocytic superoxide radicals	24, 25
*gtgE*	Gifsy-2	Inf	<0.001	T3SS effector GtgE; inactivates Rab32 subfamily, leading to inhibition of the phagosomal fusion with lysosome	26, 27
*sseI*	Gifsy-2	Inf	<0.001	T3SS effector *sseI*; deamidates Gα_i_ family G proteins, affecting the migration of macrophages and dendritic cells	30 – 32
*gtgA*	Gifsy-1/Gifsy-2^*c*^	72	<0.001	T3SS effector GtgA; cleaves NF-κB transcription factors RelA and RelB, suppressing proinflammatory responses	33
*sspH1*	Gifsy-1	Inf	0.015	E3 ubiquitin ligase; ubiquitinates protein kinase N1, leading to inhibition of the phagosomal fusion with lysosome	28, 29

a The odds ratio refers to the ratio of the odds that the gene is present in the HA isolates to the odds that the gene is present in the NHA isolates.

b BH-corrected *P* value of the Fisher’s exact tests.

c Homologs of *gtgA* are found in both Gifsy-1 and Gifsy-2 prophages.

### Multiple core SNPs are associated with *S.* Saintpaul isolates in HA and NHA SNP clusters, including 7 SNPs leading to disrupted genes due to PMSCs that are overrepresented among NHA isolates.

Nonsynonymous mutations have previously been associated with virulence attenuation in other nontyphoidal Salmonella serovars (18, 19). Therefore, we also identified core SNPs with alleles overrepresented among HA or NHA isolates. Among the 5,022 core SNP alleles identified, 995 led to nonsynonymous mutations (including 10 nonsense mutations), 3,457 led to synonymous mutations, and 570 led to mutations in noncoding regions in the reference genome (Data Set S5). The 10 nonsense mutations led to 7 NHA-associated and 3 HA-associated premature stop codons (PMSCs) in coding sequences (Fig. 3). Genes disrupted by NHA-associated PMSCs (Table 3) are involved in a variety of cellular processes, including carbohydrate metabolic processes (i.e., *bglA*), multidrug efflux systems (i.e., *bepF*), the type I-E CRISPR system (i.e., *ygcH*), pathogenesis (i.e., *yggR*, *ydiV*, and *stfC*), and an unknown process (i.e., *orf319*). On the other hand, HA-associated PMSCs (Table 3) were identified in genes responsible for transportation of maltose (*malX*) and sulfoacetate (*STM14_0610*), as well as a virulence gene (*avrA*) previously reported to enhance the survival of *S.* Typhimurium inside infected macrophages (20). Notably, core SNPs leading to HA and NHA-associated PMSCs tended to be associated with specific HA or NHA SNP clusters, except for *yggR* and *malX*, whose genotypic pattern correlated with lineage. Specifically, while *yggR* was disrupted in all isolates from lineage IB (representing 2 HA and 3 NHA clusters) and was intact in all isolates from lineages IA and IC (representing 3 HA and 1 NHA clusters), *malX* was intact in all isolates from lineage IB and was disrupted in all isolates from lineages IA and IC (Fig. 3).

**TABLE 3:** Genes disrupted by PMSCs overrepresented among isolates in NHA or HA SNP clusters

Gene	Odds ratio^*a*^	*P* value^*b*^	Length of intact gene (bp)	Length of disrupted gene (bp)	Gene product annotation
Genes disrupted by PMSCs^*c*^ overrepresented among NHA isolates					
* bglA*	Inf	0.003	1,434	75	6-Phospho-beta-glucosidase
* ygcH*	Inf	0.003	651	369	Type I-E CRISPR-associated protein
* bepF*	Inf	0.003	1,227	1,170	Periplasmic multidrug efflux RND transporter
* stfC*	Inf	0.003	2,658	75	Fimbrial biogenesis outer membrane usher
* yggR*	4.5	0.003	981	153	Type IV pilus twitching motility protein
* ydiV*	16.33	0.003	714	369	Anti-FlhC_2_FlhD_4_ factor
* orf319*	Inf	0.022	960	333	DUF523 and DUF1722 domain-containing protein

Genes disrupted by PMSCs overrepresented among HA isolates					
* malX*	0.22	0.003	237	144	PTS maltose transporter subunit IIBC
* STM14_0610*	0	0.003	1,248	321	MFS transporter; putative sulfoacetate transporter SauU
* avrA*	0	0.003	867	48	SPI-1 encoded acetyltransferase

a The odds ratio refers to the ratio of the odds that the gene is intact among the HA isolates to the odds that the gene is intact among the NHA isolates.

b BH-corrected *P* value of the Fisher’s exact tests.

c PMSCs, premature stop codons.

10.1128/mSphere.00730-21.9 DATA SET S5 Statistics of core SNPs that have alleles significantly over- or underrepresented among HA/NHA isolates. Download Data Set S5, XLSX file, 0.6 MB. Copyright © 2022 Chen et al. 2022 Chen et al. https://creativecommons.org/licenses/by/4.0/ This content is distributed under the terms of the Creative Commons Attribution 4.0 International license.

### The ability of *S.* Saintpaul strains to invade human intestinal epithelial cells varies across strains representing different lineages and is independent of their association with HA/NHA SNP clusters.

As the ability of NTS to invade cells in the intestinal epithelium is central to the proinflammatory response that Salmonella uses to generate a novel nutrient niche, allowing it to compete with the resident gut microbiota (21), we initially characterized the ability of *S.* Saintpaul strains representing 4 HA and 3 NHA SNP clusters to invade human intestinal epithelial (HIEC-6) cells. The invasion efficiency was quantified as the recovery rate, which was defined as the ratio of the number of salmonellae recovered 1 h postinfection (hpi) to the number of salmonellae used for infection. Among the strains included in the phenotypic experiments, the recovery rate ranged from 0.01 (FSL R9-7540; PDS000006321) to 0.04 (FSL R9-7866; PDS000032614) for strains representing HA SNP clusters and from 0.01 (FSL R12-1550; PDS000004385) to 0.05 (FSL R12-1548; PDS000004383) for strains representing NHA SNP clusters.

**FIG 4:**
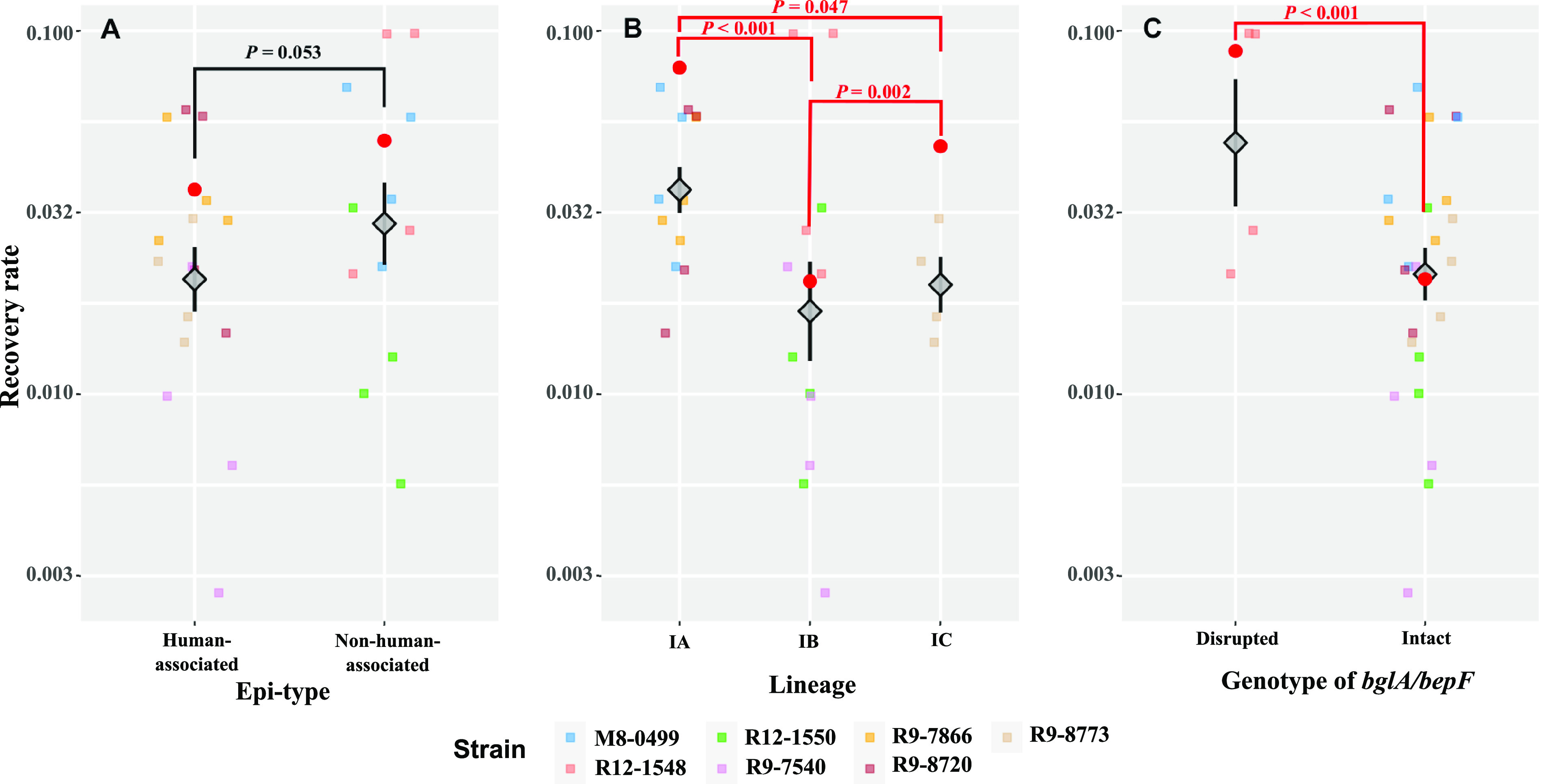
Lineage and genotype of *bglA*/*bepF*, but not epi-type, are associated with the differences in the ability of *S.* Saintpaul strains to invade HIEC-6 cells. HIEC-6 cells were infected by representative strains of *S.* Saintpaul (MOI, 100 per cell), followed by incubation at 37°C with 5% CO_2_ for 1 h. Extracellular salmonellae were killed by treating the infected HIEC-6 cells with tissue culture medium containing 20 μg/mL gentamicin, followed by a 1-h incubation at 37°C with 5% CO_2_. The invasion efficiency is represented by the recovery rate, defined as the ratio of the number of bacterial cells that successfully invaded the HIEC-6 cells to the number of bacterial cells used for infection. All data points of recovery rates are shown, in addition to the means (diamonds) ± standard errors (error bars) across biological replicates and strains for each epi-type (A), lineage (B), and genotype of *bglA*/*bepF* (C). The estimated marginal means of recovery rate (RREMM) based on the linear regression model (the INV model) (Table 4) are shown as red dots. *Post hoc* comparisons of the RREMM were performed between strains (i) from different lineages (Tukey’s honestly significant difference test), (ii) with different genotypes of *bglA*/*bepF* (*t* test), and (iii) from SNP clusters classified as different epi-types (i.e., HA or NHA; *t* test). *P* values for each of the comparisons are presented (red, *P < *0.05; black, *P ≥ *0.05).

Using the “best subset selection” method, the linear regression model that allowed for the best prediction of the invasion efficiency of the representative *S.* Saintpaul strains (the INV model) included epi-type, lineage, and the genotype of *bglA*/*bepF* as fixed effects (Table 4 and Fig. 4). Lineage had a significant effect on invasion efficiency (*P < *0.001), suggesting that there were differences in invasion efficiency across *S.* Saintpaul from the 3 different lineages represented among the strains tested. *Post hoc* pairwise comparisons revealed that the invasion efficiency was significantly higher for lineage IA strains (recovery rate based on estimated marginal means [RREMM], 0.08) than for lineage IB (RREMM, 0.02; *P < *0.001) and IC (RREMM, 0.05; *P = *0.047) strains and for lineage IC strains than lineage IB strains (*P = *0.002) (Fig. 4B). The *bglA*/*bepF* genotype was also identified as a significant main effect (Table 4); the invasion efficiency was significantly higher (*P < *0.001) for strains in which these genes were disrupted (RREMM, 0.09) than for strains in which these genes were intact (RREMM, 0.02) (Fig. 4C); however, it is noteworthy that the disruption of these genes was identified only in isolates from the NHA SNP cluster PDS000004383. While epi-type was identified as a significant main effect (Table 4), the RREMM was higher for the strains representing NHA SNP clusters (0.05) than HA SNP clusters (0.04) and the *post hoc t* test suggested that this difference in invasion efficiency was only marginally significant (*P = *0.053) (Fig. 4A). Overall, our results suggest that the ability of *S.* Saintpaul to invade human intestinal epithelial cells is not linked to the association of *S.* Saintpaul HA SNP clusters with human clinical cases.

**TABLE 4:** One-way ANOVA statistics for linear regression models^*a*^ for invasion efficiency and intracellular survival of *S.* Saintpaul strains

Model and factor	df^*b*^	Sum sq^*c*^	Mean sq^*d*^	*F* value^*e*^	*P*r (>*F*)^*f*^
INV model					
* *Biological replicate	3	1.27	0.42	21.17	<0.001
* *Epi-type	1	0.16	0.16	8.01	0.010
* *Lineage	2	0.99	0.50	24.75	<0.001
* bglA*/*bepF*^*g*^	1	0.92	0.92	45.84	<0.001
					
ICS-1 model					
* *Biological replicate	2	0.26	0.13	3.42	0.060
* *Lineage (IC vs IA/IB)^*h*^	1	0.32	0.32	8.45	0.011
* bglA*/*bepF*^*g*^	1	0.15	0.15	4.05	0.063
* ygcH*/*stfC*^*g*^	1	0.11	0.11	2.87	0.111
					
ICS-2 model					
* *Biological replicate	2	0.17	0.08	7.80	0.004
* *Lineage	2	0.12	0.06	5.46	0.016
					
ICS-3 model					
* *Biological replicate	2	0.84	0.42	11.77	<0.001
* sodCI*	1	0.51	0.51	14.17	0.002

a The models were fitted (using reference coding [R default]) to the data for invasion efficiency (the INV model), as well as intracellular survival between 0 and 2 hpi (the ICS-1 model), 2 and 6 hpi (the ICS-2 model), and 6 and 24 hpi (the ICS-3 model).

b df, degree of freedom.

c Sum sq, sum of squares due to the factor.

d Mean sq, mean of the sum of squares due to the factor.

e *F* value, *F* statistic.

f *P*r (>*F*), *P* value of the *F* test.

g Genomic signatures grouped together due to identical genotypic patterns across *S.* Saintpaul strains.

h Strains representing lineages IA and IB were grouped together and compared with strains representing lineage IC, as feature selection suggested that only the dummy variable for lineage IC should be included in the final model.

### Differences in the ability of *S.* Saintpaul strains to survive in macrophage-like cells suggest that lineage and the presence/absence of *sodCI* affect intracellular survival.

Besides the ability to attach and invade human intestinal epithelial cells, survival and replication in macrophages represent another infection-relevant process associated with systemic infection (22–25). In addition, our comparative genomic analyses identified an HA gene (*sodCI*), which encodes a copper/zinc co-factored (Cu-Zn) superoxide dismutase that has been previously shown to contribute to Salmonella intracellular survival in macrophages (26, 27). Therefore, we characterized the change in intracellular levels of *S.* Saintpaul strains in macrophage-like cells between 0 and 2, 2 and 6, and 6 and 24 hpi to identify associations between resistance to macrophage killing and (i) epi-type, (ii) lineage, and (iii) relevant genomic signatures (defined as presence/absence of HA virulence genes and HA/NHA-associated core SNPs leading to PMSCs). Intracellular survival during different time periods (i.e., 0 to 2, 2 to 6, and 6 to 24 hpi) was quantified as the differences in the log_10_-transformed level (CFU/mL) of the *S.* Saintpaul strains recovered from human macrophage-like cells. During the 0 to 2 hpi time period, all strains decreased in level with respect to the initial level used for infection; the differences in log_10_ CFU/mL ranged from −1.07 (FSL R9-8720; PDS000004371) to −0.65 (FSL R9-8773; PDS000002536) for strains representing HA SNP clusters and from −1.18 (FSL R12-1550; PDS000004385) to −0.79 (FSL R12-1548; PDS000004383) for strains representing NHA SNP clusters. During the 2 to 6 hpi time period, an increase in level was observed for all strains, ranging from 0.12 (FSL R9-8720; PDS000004371) to 0.34 (FSL R9-8773; PDS000002536) for strains representing HA SNP clusters and from 0.21 (FSL M8-0499; PDS000029303) to 0.32 (FSL R12-1550; PDS000004385) for strains representing NHA SNP clusters. Finally, during the 6 to 24 hpi time period, the *S.* Saintpaul strains tended to either maintain or show a decrease in their intracellular levels; the differences in log_10_ CFU/mL for this time period ranged from −0.35 (FSL R9-8773; PDS000002536) to −0.01 (FSL R9-7866; PDS000032614) for strains representing HA SNP clusters and from −0.50 (FSL R12-1548; PDS000004383) to 0.04 (FSL M8-0499; PDS000029303) for strains representing NHA SNP clusters.

**FIG 5:**
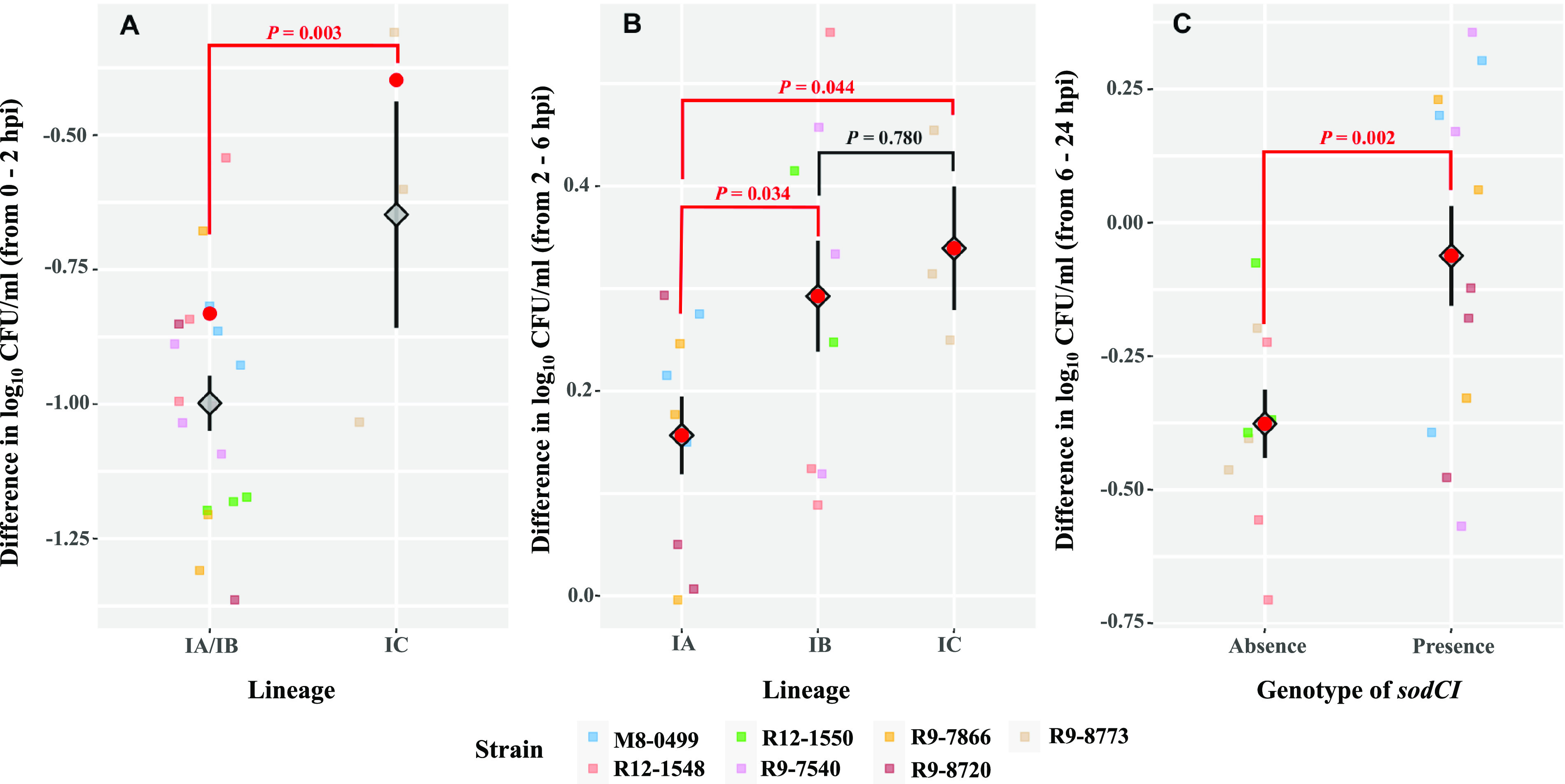
Factors associated with the survival of *S.* Saintpaul strains differ across multiple time periods after their entry into human macrophage-like cells. (A) Effect of lineage on the intracellular survival of *S.* Saintpaul strains from 0 to 2 hpi. (B) Effect of lineage on the intracellular survival of *S.* Saintpaul strains from 2 to 6 hpi. (C) Effect of presence/absence of *sodCI* on the intracellular survival of *S.* Saintpaul strains from 6 to 24 hpi. THP-1 cells were differentiated into macrophage-like cells in the presence of 20 ng/mL PMA for 3 days. Macrophage-like cells were then infected by representative strains of *S.* Saintpaul (MOI, 10 per cell), followed by incubation at 37°C with 5% CO_2_ for up to 24 h. Extracellular salmonellae were killed by treating the infected macrophage-like cells with tissue culture medium containing 20 μg/mL gentamicin, followed by incubation at 37°C with 5% CO_2_ for the indicated time periods. The intracellular survival within a given time period is represented by the difference in log_10_ CFU/mL, calculated by subtracting the log_10_ CFU/mL at the beginning from the log_10_ CFU/mL at the end of the time period. All data points of the difference in log_10_ CFU/mL are shown, in addition to the means (diamonds) ± standard errors (error bars) across biological replicates and strains for different lineages between 0 and 2 hpi (A) and 2 and 6 hpi (B) and for different genotypes of *sodCI* between 6 and 24 hpi (C). The estimated marginal means of the difference in log_10_ CFU/mL (LDEMM) based on the linear regression models (the ICS-1, ICS-2, and ICS-3 models) (Table 4) are shown as red dots. *Post hoc* comparisons of the LDEMM were performed between strains from different lineages (Tukey’s honestly significant difference test and *t* test) and with different genotypes (*t* test). *P* values for each of the comparisons are presented (red, *P < *0.05; black, *P ≥ *0.05).

Separate linear regression models were constructed for each time period (the ICS-1, ICS-2, and ICS-3 models for 0 to 2, 2 to 6, and 6 to 24 hpi, respectively) to assess the impact of epi-type, lineage, and relevant genomic signatures on the survival of *S.* Saintpaul strains in macrophage-like cells within a given time period. Different factors were retained in the models as fixed effects, including (i) lineage (comparison between IC and IA/IB only), the *bglA*/*bepF* genotype, and the *ygcH*/*stfC* genotype for the ICS-1 model, (ii) lineage for the ICS-2 model, and (iii) the *sodCI* genotype for the ICS-3 model (Table 4). Between 0 and 2 hpi, the strain representing lineage IC showed a significantly lower reduction (i.e., with respect to the initial inoculation; estimated marginal mean of the difference in log_10_ CFU/mL [LDEMM], −0.40) than the strains representing lineages IA and IB (LDEMM, −0.83) (Table 4 and Fig. 5A). Notably, *sspH1*, a Gifsy-1-associated virulence gene encoding an E3 ubiquitin ligase, was found only in the lineage IC isolates (represented by a single HA SNP cluster, PDS000002536). Therefore, further investigations of this gene, among other genomic signatures specific for lineage IC, may provide insights into the survival mechanisms of *S.* Saintpaul upon entry into macrophage-like cells. In contrast to the results for invasion efficiency, the intracellular growth between 2 and 6 hpi was significantly lower for strains representing lineage IA (LDEMM, 0.16) than for strains representing lineages IB (LDEMM, 0.29) and IC (LDEMM, 0.34). Finally, virulence gene *sodCI*, which was present in 4 HA and 1 NHA SNP clusters, was significantly associated with differences in intracellular survival between 6 and 24 hpi (Table 4 and Fig. 5C). Strains with *sodCI* (LDEMM, −0.06) showed a significantly higher ability to survive in macrophage-like cells between 6 and 24 hpi than strains without this gene (LDEMM, −0.38).

For all three time periods (0 to 2, 2 to 6, and 6 to 24 hpi), no differences in intracellular survival or growth were identified between strains representing HA and NHA SNP clusters, suggesting that the strains representing NHA SNP clusters did not show a consistent deficiency in their ability to resist macrophage killing or grow in macrophages in comparison to HA SNP clusters.

## DISCUSSION

The Healthy People 2020 initiative aimed to reduce the incidence of clinically diagnosed nontyphoidal salmonellosis in the United States to 11.4 cases per 100,000 population per year (https://www.healthypeople.gov/2020/topics-objectives/topic/food-safety) from a benchmark of 15.0 per 100,000 population for 2006 to 2008; in 2019, the preliminary incidence reported was 17.1 cases per 100,000 people (11), suggesting that despite ongoing efforts across government and industry to reduce the incidence of salmonellosis, these efforts are not currently achieving the desired reduction in human clinical cases. We therefore proposed a novel framework for reprioritizing control efforts to focus on Salmonella subtypes that are most likely to result in human clinical illness (10). In this study, we illustrate this approach by using genomic analyses based on existing WGS and associated metadata available in the NCBI PD database, followed by phenotypic experiments, to identify genomic signatures that are associated with (i) Salmonella subtypes that show significantly higher or lower proportions of human clinical isolates as well as (ii) phenotypes that reflect Salmonella behavior under infection-relevant conditions. While tissue culture experiments failed to identify consistent phenotypic characteristics indicative of enhanced human virulence of HA strains, identification of a candidate gene (i.e., *sodCI*) overrepresented among HA isolates and associated with enhanced intracellular survival in human macrophage-like cells illustrates the potential of the overall approach used here and is consistent with prior data suggesting specific genomic signatures for enhanced or reduced human virulence of nontyphoidal Salmonella. Identification of multiple virulence factors (including *sodCI*) that merit further assessments for understanding their role in human clinical salmonellosis also indicates that the approach used here can be valuable for identifying putative Salmonella virulence-associated genomic signatures across a wide range of serovars.

### While *S.* Saintpaul includes SNP clusters that show strong associations with human and nonhuman isolation sources, universal genomic or phenotypic signatures associated with human hypo- or hypervirulence are difficult to identify.

Overall, we identified 29 and 23 SNP clusters that showed significant over- and underrepresentation of human clinical isolates, respectively. While it is likely that some if not many of these SNP clusters represent clonal groups with an enhanced or reduced ability to cause human diseases, a subset of these SNP clusters may show an over- or underrepresentation of human clinical isolates due to biases in isolate collection and deposition into the NCBI PD database. However, we have manually inspected the SNP clusters to identify and preclude SNP clusters with obvious biases, such as the inclusion of a large number of human isolates that likely represented a single outbreak or a large number of environmental isolates that were derived from a single source population (e.g., a single farm or possibly a single processing facility). Importantly, HA and NHA SNP clusters were diverse and did not represent one or a few distinct lineages within *S.* Saintpaul, suggesting that there is not a single monophyletic *S.* Saintpaul phylogenetic group representing human or environmentally adapted strains. In contrast, specific host or environmentally adapted monophyletic Salmonella lineages have been described for other Salmonella serovars. For example, *S.* Kentucky ST152 represents a specific monophyletic lineage that appears to show reduced human virulence (28). On the other hand, existence of multiple, nonmonophyletic groups that show unique virulence characteristics have also been described, such as DT104 and DT193, two distinct multidrug-resistant *S*. Typhimurium clonal groups (29, 30), or a large number of distinct Listeria monocytogenes isolates (representing multiple serotypes) that carry different PMSCs in the virulence gene *inlA* (31, 32). The conclusion that *S.* Saintpaul may include multiple distinct clades that are likely to show enhanced or reduced ability to cause human infections is also supported by the observation that we did not identify unifying genomic signatures or phenotypic characteristics among the “high-confidence” HA or NHA clusters that were further investigated here (as discussed further below). It is important to note that we restricted our analyses to examining the virulence potential in humans and that some of the NHA isolates identified may retain the ability to cause disease in other animals. Regardless, our findings illustrate the challenges for defining hypo- and hypervirulent Salmonella isolates across diverse serovars and potential limitations of phenotypic assays when evaluating human virulence.

### Multiple virulence genes and core SNPs leading to PMSCs are associated with the over- or underrepresentation of HA/NHA SNP clusters among human clinical isolates, including genes that may be responsible for an elevated ability to resist macrophage killing.

Our characterizations of *S.* Saintpaul suggested that isolates in HA SNP clusters carried virulence genes (i.e., *gtgA*, *sodCI*, *gtgE*, *sseI*, and *sspH1*) associated with prophages Gifsy-1 and Gifsy-2, which were largely absent from NHA SNP clusters. NTS serovars are known to carry a variety of prophages (13), including Gifsy-1 and Gifsy-2, two of the most well-studied Salmonella prophages. These prophages have previously been reported to encode virulence factors that facilitate Salmonella’s ability to (i) resist killing by immune cell-derived superoxide radicals via the activity of SodCI (26, 27), (ii) prevent phagolysosomal fusion to escape lysosome degradation via the activity of GtgE and SspH1 (33–36), (iii) interfere with the migration of macrophages and dendritic cells via the activity of SseI (37–39), and (iv) inhibit the NF-κB signaling pathway to dampen proinflammatory responses via the activity of GtgA (40). In the host, these abilities collectively result in dampening of the host immune response, allowing Salmonella to fine-tune the proinflammatory response needed for procuring a novel nutrient niche while delaying clearance by the host immune system.

Previous studies have documented a role for SodCI at both cellular and organismal levels, including a study that indicated that SodCI was essential for Salmonella resistance to reactive oxygen species produced by phagocytic cells (26). Golubeva and Slauch furthermore showed that *sodCI* transcription was highly induced in both macrophage cell lines and in the spleens of Salmonella-infected mice (27). Our phenotypic analyses suggested that the presence of *sodCI* was significantly associated with improved intracellular survival of representative *S.* Saintpaul strains in macrophage-like cells between 6 and 24 hpi but not for earlier time points; this finding is consistent with our data showing the overrepresentation of *sodCI* presence among HA SNP cluster strains. Additionally, our results showed an enhanced ability of a lineage IC strain to survive in macrophage-like cells between 0 and 2 hpi, which might be attributed to the presence of *sspH1* only in this strain. Previous studies have shown that SspH1 ubiquitinates host PKN1 (35), allowing for sustained activation of Akt kinase and thus inhibition of the phagolysosomal fusion (36). While the enhanced intracellular survival of the lineage IC strain tested here may be associated with the presence of *sspH1* in lineage IC isolates (and absence of this gene in all other isolates), additional studies (including assessments of null mutants) will be needed to confirm a role for *sspH1* in intracellular survival in macrophages. The remaining Gifsy-1/2-borne virulence genes (i.e., *sseI*, *gtgE*, and *gtgA*) were not identified as factors significantly affecting the virulence-associated phenotypes tested here. This may be due to (i) a lack of a sufficient number of appropriate strains available for phenotypic testing (e.g., only 1 of the 7 strains included harbors *sseI* and *gtgE*), (ii) strain genotypes that showed strong correlation of presence/absence patterns for multiple genes (i.e., *gtgA* was significantly correlated with *sodCI*, which showed a higher *R* value with respect to the responses), (iii) differences in gene expression of these loci, and/or (iv) inability of tissue culture assays to assess all phenotypes relevant to human virulence.

In addition to the presence of virulence genes, nonsynonymous mutations in virulence and stress response genes have previously been associated with virulence attenuation in other NTS serovars (18, 41, 42). For example, previous studies on a swine-adapted *S.* Derby lineage identified a nonsynonymous mutation leading to a PMSC in *hilD*, which causes the loss of function of the master regulator (HilD) of the Salmonella pathogenicity island 1 (SPI-1) and an associated reduced ability to invade human epithelial cells (41). In our study, all representative isolates from one NHA SNP cluster harbored a PMSC in *stfC*, encoding a fimbrial usher, while this gene was intact in all isolates in the HA SNP clusters. Fimbriae have been suggested to mediate the binding of Salmonella to different host cell surfaces (43–46). Although inactivation of Stf fimbria in *S.* Saintpaul may contribute to the observed low proportions of human isolates in this SNP cluster, further studies characterizing the role of this fimbria in virulence are needed, as previous studies suggest that inactivation of this gene cluster may have negligible effects *in vivo* (47).

Together, the combination of genes and core SNPs associated with enhanced invasion efficiency or intracellular survival support a model in which multiple genomic signatures may be associated with Salmonella fitness under different infection-relevant conditions. Therefore, it is likely that the collection of virulence factors, rather than the identification of a single virulence factor, will be informative for understanding the observed overrepresentation of certain *S.* Saintpaul subtypes among human clinical cases.

### Invasion of human intestinal epithelial cells and survival in macrophage-like cells alone do not represent phenotypic characteristics that are consistent with the high and low proportions of human clinical isolates observed among HA/NHA SNP clusters.

Although invasion and intracellular survival within immune cells, such as macrophages, are important aspects of the pathogenesis of nontyphoidal Salmonella, they represent only a fraction of the host-Salmonella interactions that occur during an infection (21, 47–50). Our characterizations using infection of epithelial and macrophage cell models suggest that the two phenotypic assays used here cannot fully explain the association of SNP clusters with human clinical isolates. Collectively, NTS uses a suite of virulence factors during an infection to (i) mobilize in the gastrointestinal tract (51, 52), (ii) attach to the intestinal epithelial cells (53), (iii) outcompete the commensal microbiota for nutrients (54, 55), (iv) utilize alternative electron acceptors produced during inflammation (56, 57), and (v) interfere with the migration of immune cells (39). For example, among the virulence genes overrepresented among HA SNP clusters, *sseI* encodes a protein that affects the motility and migration of immune cells (37, 39) and *gtgA* encodes a protease that inactivates the NF-κB signaling pathway leading to proinflammatory responses (40). However, neither the immune cell motility nor the proinflammatory responses can be assessed using the experiments performed here. Interestingly, our results indicated that lineage IA strains showed an elevated invasion efficiency but an attenuated intracellular growth between 2 and 6 hpi in comparison to the strains representing lineages IB and IC. This illustrates another challenge with tissue cultures assays—results across different phenotypic characterizations do not necessarily support a singular clear conclusion of enhanced or reduced virulence. It is important to note, however, that tissue culture assay results do not necessarily always translate to virulence in a whole animal or human in the most apparent way; for example, enhanced cytotoxicity in a tissue culture model may translate to more rapid clearance in a host and hence reduced virulence (58). Overall, our results reflect the well documented complex nature of Salmonella’s interactions with the host during the course of an infection and suggest that the use of additional model systems, such as mouse models, are necessary to better understand which genomic signatures are associated with the overrepresentation of human clinical isolates among some subtypes of *S.* Saintpaul.

### Improved surveillance data and biological characterizations of virulence factors in a greater diversity of serovars are needed for a risk-based classification of Salmonella.

Our understanding of salmonellosis, and more specifically the roles of various genes, has been informed primarily by characterizations of the two model serovars, *S.* Typhi and *S.* Typhimurium, representing models for typhoidal and nontyphoidal salmonellosis, respectively. Furthermore, only a handful of strains have been used as models for each (e.g., *S.* Typhi strains CT18 and Ty2 and *S*. Typhimurium strains LT2, 14028S, and SL1344). Importantly, genomic characterizations in other nontyphoidal serovars are essential for developing a framework that focuses on the presence of specific genomic signatures, rather than serotyping, to guide risk-based control efforts focusing on Salmonella subtypes that are more frequently associated with human clinical disease.

Another key challenge in implementing the approach outlined here is developing a standardized set of criteria for ensuring the availability of accurate and complete metadata for isolates included in genomic analyses. Currently, the WGS data uploaded to the NCBI PD database have limited accompanying metadata, such as whether the isolate is associated with a human or nonhuman source. In particular, human clinical isolates lack information on the date and location of collection, due primarily to confidentiality reasons. Conversely, nonhuman isolates tend to be associated with more complete associated metadata. However, there is currently no standardization for how dates are reported (i.e., some include year, others year and month, and others year, month, and day), and if a geographical location is provided, there is no standardization for what level (i.e., country versus state/province) these data are provided; similar challenges may be observed for isolation source, as “environmental/other” is used to capture all isolates that are not from human clinical cases, but they may still represent isolates causing animal clinical infections. In our study, we used a combination of year of isolation, geographic location, and source, in addition to SNP distances, to ensure that comparisons were based on a sufficiently diverse collection of isolates. However, this proved challenging, as a number of metadata terms were missing or provided incomplete records. Therefore, standardization of metadata is important for the implementation of the approach described here.

In conclusion, we used *S.* Saintpaul as a model to implement and test a proposed framework, which leverages WGS and associated metadata available in the NCBI PD database, as well as phenotypic experiments to identify and study genomic and phenotypic characteristics that may be responsible for human hypo- or hypervirulence. While our findings illustrate the challenge of identifying and characterizing human hypo- or hypervirulent Salmonella strains across different nontyphoidal Salmonella serovars, our experiments were able to identify virulence genes and core SNPs leading to PMSCs associated with NCBI PD SNP clusters with over- or underrepresentation of human clinical isolates, including genes (e.g., *sodCI*) that were associated with an enhanced ability to survive in human macrophage-like cells. Further development of this framework, which ultimately can be used to predict and quantify virulence potential for a risk-based approach to controlling Salmonella in the food supply, may need to include a focus on (i) using additional phenotypic approaches (e.g., animal models) to assess virulence characteristics (although these experiments can be challenging, as it is possible that strains with attenuated human virulence still show full virulence in an animal model), (ii) constructing and characterizing appropriate mutants to mechanistically establish the role of genomic signatures associated with human and environmentally associated SNP clusters and clonal groups, and (iii) improving metadata availability for Salmonella isolates deposited in the NCBI PD database.

## MATERIALS AND METHODS

### Selection of *S.* Saintpaul.

Metadata for the 100 SNP clusters of Salmonella enterica subsp. *enterica* (abbreviated “*S.*”) serovars having the greatest number of isolates in the NCBI pathogen detection (PD) database (https://www.ncbi.nlm.nih.gov/pathogens/) were first assessed (accessed 20 February 2020) to determine the proportion of isolates in each SNP cluster with the source type “clinical” (i.e., isolated from human clinical sources) (see Data Set S1, tab 2, in the supplemental material). *S.* Saintpaul was selected as the model serovar for use in this study because (i) *S.* Saintpaul has SNP clusters with high and low proportions of human clinical isolates, suggesting that some SNP clusters may be more likely to be associated with human clinical illness (Data Set S1, tab 2), (ii) the public health relevance associated with this serovar has been increasing for the past 15 years, and (iii) sufficient metadata and associated whole-genome sequence (WGS) data were available for this serovar in the NCBI PD database for conducting statistical and genomic analyses.

### Phylogenetic analyses.

Phylogenetic analyses were performed to assess whether *S.* Saintpaul is polyphyletic (i.e., comprising isolates with identical antigenic formulae from distinct lineages that do not share the same most recent common ancestor). Specifically, from each SNP cluster, the isolate whose genome assembly had the highest *N*_50_ value and/or the lowest number of contigs was selected as the representative isolate for the given SNP cluster. Genome assemblies of the representative isolates of the SNP clusters were downloaded from the NCBI PD database, followed by using SISTR v1.0.2 (59) to confirm the reported serotype (i.e., I 1,4,[5],12:e,h:1,2). To comprehensively assess the phylogeny of *S.* Saintpaul, a diverse collection of reference isolates was included in the phylogenetic analysis; these reference isolates, selected based on their inclusion in previous data sets defining the phylogenetic structure of Salmonella enterica (13, 60), represent (i) 313 unique Salmonella enterica subsp. *enterica* serovars and one serovar each for an additional five Salmonella enterica subspecies and (ii) serovars associated with human and animal clinical salmonellosis. Core SNPs among the representative isolates of *S.* Saintpaul SNP clusters and the reference isolates were identified using kSNP3 v 3.1 (61) with an optimal k-mer size of 19 estimated by Kchooser. RAxML (62) was used to construct maximum likelihood phylogenetic trees based on alignments of core SNPs, specifying the GTRCATX model (63, 64) with Lewis ascertainment bias correction (65) and 1,000 bootstrap replicates. The phylogenetic trees were visualized and edited with the Interactive Tree of Life (iTOL) (66). K-mer-based identification of core SNPs was selected instead of core genome multilocus sequence typing (cgMLST) because the former generates an SNP matrix that can be used to construct a phylogeny using a maximum likelihood method (a probabilistic method) while cgMLST generates a matrix of allelic differences (i.e., genetic distance) that can only be used to generate trees using distance-based methods, such as the unweighted pair group method with arithmetic mean and neighbor-joining.

### Association assessment and selection of SNP clusters.

For each *S.* Saintpaul SNP cluster, the association with human clinical salmonellosis cases was assessed by calculating an odds ratio (i.e., the odds of “human clinical” isolates that were assigned to the SNP cluster divided by the odds of “human clinical” isolates that were not assigned to the SNP cluster), followed by a one-sided Fisher’s exact test (with the Benjamini-Hochberg [BH] correction for multiple testing) for determining statistical significance. SNP clusters were classified into different epidemiology types (epi-types) based on the proportion of human clinical isolates among all isolates assigned to them. SNP clusters with significantly high or low proportions of human clinical isolates were considered human-associated (HA) and non-human-associated (NHA) SNP clusters, respectively, while the other SNP clusters were not assigned an epi-type. Ten SNP clusters (*n* = 5 for each of HA and NHA SNP clusters) were selected for use in the comparative genomic analyses and the phenotypic characterizations based on (i) the significance of the BH-corrected *P* values (i.e., significance of the Fisher’s exact tests), (ii) the availability of genomic assemblies for at least 50 isolates in the SNP cluster, and (iii) multiple geographic locations and collection (or creation) years associated with the isolates in the SNP clusters (to exclude SNP clusters representing isolates likely associated with large outbreaks or environmental assessment events).

### Selection of isolates, assessment of phylogenetic relationship, and genome annotation.

A total of 10 isolates were selected from each selected HA and NHA SNP cluster (designated HA and NHA isolates, respectively) to represent the genomic diversity of all isolates assigned to the SNP cluster. Specifically, isolates assigned to each SNP cluster were ranked based on the min-same (minimum SNP distance from the isolate to another isolate in the SNP cluster with the same source category) and min-diff (minimum SNP distance from the isolate to another isolate in the SNP cluster from the opposite source category) values from highest to lowest, followed by calculating the average of the two ranks (average rank). The isolates with the lowest average ranks (i.e., highest average diversity) were prioritized. For each of the 10 SNP clusters, 10 isolates were selected to represent (i) the lowest average rank, (ii) a diversity of geographical location, and (iii) a range of years of collection (Data Set S2, tab 2).

Genome assemblies of the selected *S.* Saintpaul isolates representing each of the selected SNP clusters, if available, were downloaded from the NCBI PD database. For the isolates whose assemblies were not available, raw data were retrieved from the NCBI Sequence Read Archive (https://www.ncbi.nlm.nih.gov/sra/), checked for quality using FastQC v0.11.8 (67), and *de novo* assembled using SKESA v2.4.0 (68). SISTR v1.0.2 (59) was used to confirm the serotype of all isolates. To assess the phylogenetic relationship among the isolates, kSNP3 v3.1 (61) was used to identify core SNPs among the isolates (k-mer size of 19 was used), along with two isolates representing serovars Coeln and Haifa added to the analysis as outgroups; a matrix showing the pairwise core SNP differences was created using Geneious Prime 2020.2.2. A maximum likelihood phylogenetic tree (GTRCATX model with Lewis ascertainment bias correction; 1,000 bootstrap replicates) based on the core SNPs was constructed using RAxML v8.2.12 (62); the tree was visualized and edited using iTOL (66).

Genome assemblies of the selected *S.* Saintpaul isolates were annotated in general feature format (GFF3) using Prokka v1.14.5 (69) with standard settings for Gram-negative organisms.

### Identification of HA and NHA genes.

Panaroo v1.2.3 (70) was used to infer the pangenome among the selected *S.* Saintpaul isolates. Gene sequences were clustered into putative families with a threshold of 70% sequence identity; the pangenome extraction was checked using rarefaction curves (Fig. S2B). To identify genes that may contribute to the human virulence of *S.* Saintpaul, the pangenome-wide association study (pan-GWAS) was first performed using Scoary v1.6.14 (71) to identify genes whose presence is over- or underrepresented among HA/NHA isolates; the statistical significance was assessed using Fisher’s exact tests with BH correction for multiple testing. Subsequently, the genes with statistical significance (BH-corrected *P* value < 0.05) were further annotated using InterProScan v5.44-79.0 (72) to retrieve additional annotations for the genes annotated by Prokka as encoding hypothetical proteins. The gene annotations were manually inspected for functions that may contribute to the human virulence of *S.* Saintpaul. To facilitate the identification of genes of interest based on genome annotation, the –collapse flag was not specified while running Scoary.

### Identification of HA and NHA core SNPs.

A k-mer-based SNP variant calling was performed using kSNP3 v3.1 (61) to identify core SNPs among the selected *S.* Saintpaul isolates. The -vcf flag was specified, and the genome assembly (GCA_007021805.1) of one isolate (PDT000217923.2) from an HA SNP cluster (PDS000006321) was designated the reference in the program for core SNP annotation, as this genome assembly had the lowest number of contigs (18 contigs). Core SNPs in protein-coding genes were categorized into synonymous and nonsynonymous mutations (i.e., missense and nonsense mutations). To identify core SNPs that have alleles overrepresented among isolates assigned to HA/NHA SNP clusters, pan-GWAS was performed using the same method as described for the gene presence/absence analysis. In cases where a core SNP had more than one alternative allele (i.e., alleles other than the reference allele), each alternative allele was assessed separately. Genes disrupted by a premature stop codon (PMSC) that were overrepresented among HA/NHA isolates were screened out by manually inspecting the annotation of the core SNPs.

### Identification of plasmid-borne genes overrepresented among isolates in HA or NHA SNP clusters.

For each assembly, contigs were classified into putative chromosome or plasmid sequences using Platon (73–76), followed by a series of homology searches using BLAST+ (77). Specifically, *in silico* identification of putative plasmid sequences was first performed using Platon; through the program, a plasmid identifier (ID) was assigned to each of these sequences if a BLAST+ search against the RefSeq plasmid sequence database (78–80) indicated significant matches (i.e., matches with ≥80% query coverage and ≥80% percentage sequence identity). The putative plasmid sequences without significant matches were additionally searched against the PATRIC plasmid sequence database (81) using BLAST+ with the same criteria, and a plasmid ID was assigned to each sequence for which significant matches were found. To increase the sensitivity of the *in silico* identification of putative plasmid sequences, the genome assemblies of the plasmids assigned to one or more of the putative plasmid sequences were downloaded from the NCBI website (https://www.ncbi.nlm.nih.gov/) and searched against the genome assembly of the isolate via BLAST+. Plasmids with ≥80% query coverage and ≥80% percentage sequence identity were considered “candidate plasmids” present in the genome of the isolate. Candidate plasmids were manually inspected to identify groups of plasmids that match to the same regions in the genome assembly, in which cases only the one with the best match (i.e., highest query coverage and/or percentage sequence identity) was retained for further analyses. For a specific isolate, a contig was considered plasmid-borne if (i) it was identified by Platon as a putative plasmid sequence or (ii) it matched to the candidate plasmids with ≥80% total sequence coverage and percent identity. Finally, to enumerate plasmid-borne genes overrepresented among isolates assigned to HA/NHA SNP clusters, indices of the genes assigned by Prokka were matched to the contigs, and the total number of the genes that were matched to the plasmid-borne contigs were enumerated.

### Identification of prophage-borne genes overrepresented among isolates in HA or NHA SNP clusters.

For each assembly, putative prophage sequences in the genome assembly were identified using PHASTER (82, 83). The contigs and the associated coordinates were extracted for (i) the putative prophage sequences and (ii) the genes overrepresented among isolates assigned to HA/NHA SNP clusters, followed by mapping the genes to the putative prophage sequences. The total number of genes that fell within the regions of putative prophage sequences was enumerated.

### Bacterial strains, cell lines, and culture conditions.

A total of 7 *S.* Saintpaul strains, representing 4 HA (PDS000002536, PDS000004371, PDS000032614, and PDS000006321) and 3 NHA (PDS000029303, PDS000004383, and PDS000004385) SNP clusters included in the comparative genomic analyses, were selected for use in the phenotypic experimentations. The representative strains for all four HA SNP clusters and one NHA SNP cluster (PDS000029303) were selected by identifying strains associated with these SNP clusters from a collection of *S.* Saintpaul strains in the Food Microbe Tracker (https://www.foodmicrobetracker.net/) and randomly selecting one strain from each of these SNP clusters. *S.* Saintpaul strains associated with an additional two NHA SNP clusters (PDS000004383 and PDS000004385) were procured from the New York State Department of Health (NYDOH), and one strain was randomly selected to represent each SNP cluster. The susceptibility of all representative strains to gentamicin was confirmed by (i) the absence of gentamicin resistance genes in their genomes and (ii) the growth inhibition observed for each strain in the presence of 20 μg/mL gentamicin (data not shown). Detailed information for the *S.* Saintpaul strains included in the phenotypic experiments is shown in Table 5.

**TABLE 5:** *S*. Saintpaul strains used in phenotypic experiments

Strain (FSL no.)^*a*^	SNP cluster	Epi-type	Isolation source	Collection date	Location	Collected by
FSL R9-8773	PDS000002536	HA	Human	Unknown	USA	CDC
FSL R9-8720	PDS000004371	HA	Human	Unknown	USA	CDC
FSL R9-7866	PDS000032614	HA	Human stool	Nov 2017	USA	CDC
FSL R9-7540	PDS000006321	HA	Human stool	Jun 2017	USA	Unknown
FSL M8-0499	PDS000029303	NHA	Jalapeno pepper	30 Jul 2008	Mexico	FDA
FSL R12-1548	PDS000004383	NHA	Ground turkey	18 Sept 2009	New York, USA	Unknown
FSL R12-1550	PDS000004385	NHA	Ground turkey	24 May 2010	New York, USA	Unknown

a Food Safety Laboratory (FSL) strain information can be found on the Food Microbe Tracker (https://www.foodmicrobetracker.net/).

Stock cultures for *S.* Saintpaul strains (Table 5) were preserved in 15% (vol/vol) glycerol at −80°C. All Salmonella strains were routinely cultured in Luria-Bertani (LB-Lennox; Difco, Detroit, MI) broth containing 5 g NaCl/L. Human intestinal epithelial cells (HIEC-6 cells) and human acute monocytic leukemia cells (THP-1 cells), both purchased from the American Type Culture Collection (ATCC), were preserved in cell culture medium supplemented with 10% (vol/vol) dimethyl sulfoxide in liquid nitrogen. Cells were routinely cultured in T75 flasks (Corning, Corning, NY) by growing the cells at 37°C with 5% CO_2_. HIEC-6 cells were cultured in Opti-MEM medium supplemented with 10 ng/μL recombinant epidermal growth factor (EGF) (Gibco-Invitrogen, Carlsbad, CA) and 10% (vol/vol) fetal bovine serum (FBS; heat inactivated) (Gibco-Invitrogen). THP-1 cells were cultured in RPMI 1640 medium supplemented with 2 mM l-glutamine, 10 mM HEPES, 0.01 mM phenol red, 1 mM sodium pyruvate, 25 mM d-glucose, 18 mM sodium bicarbonate, and 10% (vol/vol) FBS (not heat inactivated). Cell lines were routinely checked and confirmed to be free of *Mycoplasma* infection using the VenorGEM mycoplasma detection kit (Sigma-Aldrich, St. Louis, MO).

### Salmonella invasion of HIEC-6 cells.

Single colonies of Salmonella from freshly streaked plates (within 7 days) were inoculated into 5 mL LB broth, followed by incubation at 37°C and 200 rpm (New Brunswick Innova 43; Eppendorf AG, Hamburg, Germany) for 18 h. Subsequently, bacterial cultures were transferred (1 mL) into 1.5-mL Eppendorf tubes (Eppendorf AG, Hamburg, Germany), followed by centrifuging at 8,000 rpm for 5 min. Supernatants were removed, and the cell pellets were resuspended in the complete HIEC-6 cell culture medium. HIEC-6 cells were grown to 90 to 100% confluence by seeding them into a 24-well plate (4 × 10^5^ cells per well) (Corning, Corning, NY), followed by incubation at 37°C with 5% CO_2_ for 20 to 24 h. *S.* Saintpaul strains were randomly assigned a processing order for infection of HIEC-6 cells seeded in the 24-well plate. Bacterial cultures suspended in HIEC-6 cell culture medium were used to infect HIEC-6 cells at a multiplicity of infection (MOI) of 100 bacteria per HIEC-6 cell. Infected HIEC-6 cells were incubated at 37°C with 5% CO_2_ for 1 h, after which the cell culture medium was removed and the HIEC-6 cells were washed three times with 1× phosphate-buffered saline (PBS), followed by incubation with medium supplemented with 20 μg/mL gentamicin at 37°C with 5% CO_2_ for 1 h. After the 1 h of incubation, the gentamicin-containing medium was removed and the HIEC-6 cells were washed three times with 1× PBS. Subsequently, the HIEC-6 cells were detached with 0.25% trypsin-EDTA (Thermo Fisher Scientific, Waltham, MA) at 37°C for 3 min, followed by lysis with 1% Triton X-100 in 1× PBS on ice for 5 to 6 min. The HIEC-6 cell lysates were serially diluted in 1× PBS and spread-plated (100 μL) on LB agar plates in duplicate (quantification limit, 10 CFU/mL), followed by incubation at 37°C for 20 to 24 h. The bacterial inoculums were serially diluted in 1× PBS and spot-plated (20 μL) on LB agar plates in triplicate (quantification limit, 50 CFU/mL), followed by incubation at 30°C for 16 h. Colonies were enumerated with the SphereFlash automated colony counter (Neutec Group, Inc., Farmingdale, NY). Four independent experiments (biological replicates) were performed.

### Survival of Salmonella in macrophage-like cells.

Salmonella strains were grown as described for the invasion of HIEC-6 cells. Bacterial cultures were transferred (1 mL) into 1.5-mL Eppendorf tubes and diluted (1:10) once in 1× PBS. The diluted bacterial cultures were centrifuged at 8,000 rpm for 5 min, followed by removal of supernatants and resuspension of the cell pellets in the complete THP-1 cell culture medium. Prior to each experiment, THP-1 cells were seeded in three 24-well plates (6 × 10^5^ cells per well), designated for 2, 6, and 24 hpi, respectively, followed by differentiation into macrophage-like cells with treatment with 20 ng/mL phorbol 12-myristate-12-acetate (PMA) (Thermo Fisher Scientific, Waltham, MA) for 3 days at 37°C with 5% CO_2_. The condition for THP-1 cell differentiation was determined based on (i) suggestions of Starr et al. (84) and (ii) measurements of the soluble CD14 levels of differentiated THP-1 cells using ELISA (Thermo Fisher Scientific, Waltham, MA) per the manufacturer’s instructions (data not shown). *S.* Saintpaul strains were randomly assigned for infection of the PMA-differentiated THP-1 cells seeded in the 24-well plates. The PMA-differentiated THP-1 cells were infected with Salmonella at an MOI of 10 bacteria per cell for 1 h. After the 1-h infection, PMA-differentiated THP-1 cells were washed three times with 1× PBS, followed by incubation with medium supplemented with 20 μg/mL gentamicin at 37°C with 5% CO_2_ for the indicated time periods. At 2, 6, and 24 hpi, the gentamicin-containing medium was removed, and the PMA-differentiated THP-1 cells were washed three times with 1× PBS, detached with 0.25% trypsin-EDTA at 37°C for 3 min, and lysed with 1% Triton X-100 in 1× PBS on ice for 5 min. Subsequently, the PMA-differentiated THP-1 cell lysates were serially diluted in 1× PBS and spread-plated (100 μL) on LB agar plates in duplicate (quantification limit, 10 CFU/mL), followed by incubation at 37°C for 20 to 24 h. The bacterial inoculums were serially diluted in 1× PBS and spot-plated (20 μL) on LB agar plates in triplicate (quantification limit, 50 CFU/mL), followed by incubation at 30°C for 16 h. Colonies were enumerated with the SphereFlash automated colony counter. Three independent experiments (biological replicates) were performed.

### Statistical analysis.

All statistical analyses were performed in R Statistical Programming Environment v4.0.2 (85). The threshold of significance for all statistical tests was set to a *P *value of 0.05.

To identify the genotypic pattern across the *S*. Saintpaul strains used for phenotypic experiments for the virulence genes and core SNPs leading to PMSCs identified in the comparative genomic analyses (Tables 3 and 4), the genotype of these genomic signatures was determined in the genome of each strain. Specifically, the genome assemblies of the *S.* Saintpaul strains were downloaded from the NCBI PD database. Nucleotide sequences of the genomic signatures were retrieved from the Panaroo outputs and searched against the genome assemblies using BLAST+. A given gene was considered “present” in a given genome if it matched the genome assembly with ≥90% query coverage and ≥90% percentage sequence identity, and genes were considered “absent” in the genome if these criteria were not met. Additionally, the presence/absence of a PMSC was determined by manually checking the amino acid sequence alignment of the corresponding gene for an asterisk at the specific position. The genomic signatures were excluded from the downstream analyses if (i) their genotype did not vary across the *S.* Saintpaul strains (i.e., *ydiV*, *orf319*, and *STM14_0610*) or (ii) their genotypic pattern correlated with the lineage (i.e., *malX*, *yggR*, and *sspH1*). Additionally, genomic signatures with identical genotypic patterns were grouped together (e.g., *bglA*/*bepF*, *ygcH*/*stfC*, and *gtgE*/*sseI*), and each group was treated as a single factor (see Data Set S6 for the genotypic data of the genomic signatures with respect to the *S.* Saintpaul strains).

10.1128/mSphere.00730-21.10 DATA SET S6 Genotypic pattern of selected genomic signatures across *S.* Saintpaul strains included in phenotypic experiments. Download Data Set S6, XLSX file, 0.01 MB. Copyright © 2022 Chen et al. 2022 Chen et al. https://creativecommons.org/licenses/by/4.0/ This content is distributed under the terms of the Creative Commons Attribution 4.0 International license.

For the invasion assay, the invasion efficiency of the *S.* Saintpaul strains was represented by the decimal logarithm (log_10_)-transformed recovery rate log_10_ *N*/*N*_0_, where *N*_0_ refers to the number of bacterial cells used for infection and *N* refers to the number of bacterial cells recovered from the HIEC-6 cell lysates (i.e., the bacterial cells that successfully invaded). A linear regression model (designated the INV model) was constructed, using the stats package v4.0.5 (85), to (i) determine if the ability to invade HIEC-6 cells varied across *S.* Saintpaul strains representing different epi-types and lineages and (ii) identify genomic signatures (HA virulence genes and HA/NHA core SNPs leading to PMSCs) that might contribute to the ability of invasion. The response variable of the model was log_10_ *N*/*N*_0_, and the biological replicate was included as a blocking effect to account for the variation in log_10_ *N*/*N*_0_ across different biological replicates. A feature selection procedure was performed on the candidate factors, including epi-type, phylogenetic lineage, and unique genotypic patterns specific for one or multiple genomic signatures, to identify the factors that should be included in the model as fixed effects. Specifically, the correlation of each genotypic pattern with the response variable was inferred based on the *R* value, using the stats package v4.0.5 (85), and the pairwise correlation among different genotypic patterns was inferred based on the *P* value of the two-sided Fisher’s exact test (with BH correction for multiple testing) using the same package. Genotypic patterns (and thus the corresponding genomic signatures) were excluded from the model if they were correlated with other genotypic patterns with a higher *R* value. Subsequently, the “best subset selection” method, implemented with the leaps package v3.1 (86), was performed with the remaining genotypic patterns, along with epi-type and lineage, to determine the best model and thus the associated fixed effects that allowed for the best prediction of the invasion of *S.* Saintpaul strains into HIEC-6 cells based on (i) Bayesian information criterion, (ii) Mallows’ *C_p_*-statistic and (iii) adjusted *R* squared. If the three metrics suggested different models, the final model was determined by comparing the models (i) using the likelihood ratio test, implemented with the stats package v4.0.5 (85), for nested models or (ii) using cross-validation, implemented with the caret package v6.0.8 (87), for models that were not nested within each other. In cases where cross-validation indicated similar performances for the models, the model with the fewest number of fixed effects was selected to avoid potential overfitting. The effect of epi-type, lineage, and/or genomic signatures on the invasion efficiency was inferred by performing a one-way analysis of variance (ANOVA) with the final model, followed by *post hoc* comparisons (i.e., Tukey’s honestly significant difference [HSD] test and *t* test) between strains assigned to SNP clusters representing different epi-types, from different lineages, and/or with different genotypes of a given genomic signature, using the emmeans package v1.4. 8 (88).

For the intracellular survival assay, the intracellular levels of the *S.* Saintpaul strains in macrophage-like cells were represented by the log_10_-transformed CFU per mL. The difference in log_10_ CFU/mL was used to infer the ability of *S.* Saintpaul strains to survive in macrophage-like cells during different time periods, including 0 to 2, 2 to 6, and 6 to 24 hpi, and was calculated for each strain by subtracting the log_10_ CFU/mL at the beginning from the log_10_ CFU/mL at the end of each time period. Separate linear regression models were constructed for different time periods (the ICS-1, ICS-2, and ICS-3 models for 0 to 2, 2 to 6, and 6 to 24 hpi, respectively) with the difference in log_10_ CFU/mL as the response variable and biological replicate as a blocking effect. For each model, feature selection, ANOVA, and *post hoc* pairwise comparisons were performed as described for the invasion assay to identify and investigate factors, including epi-type, lineage, and genomic signatures, which had a significant impact on the survival of *S.* Saintpaul strains in macrophage-like cells.

### Data availability.

Genome assembly accession numbers for the *S.* Saintpaul representative isolates used for constructing the phylogeny and selected from the 5 HA and 5 NHA SNP clusters with the most significant association with the corresponding isolation sources are listed in Data Set S2, tabs 1 and 2, respectively. The data and codes associated with this study are available at GitHub (https://github.com/FSL-MQIP/Pew_SalmonellaSaintpaul_HumanVirulence.git). These include (i) the metadata of Salmonella enterica isolates available in the NCBI PD database (last accessed 23 June 2020), (ii) the experimental data obtained from the tissue culture assays, (iii) template codes for running BLAST+, kSNP3, Panaroo, Platon, Prokka, RAxML, Scoary, SISTR, and SKESA, (iv) codes for calculating odds ratios and performing Fisher’s exact tests, and (v) codes for statistical analyses of the tissue culture assay data.
