# Supplementary material for: Development of a Genomics-Based Approach To Identify Putative Hypervirulent Nontyphoidal Salmonella Isolates: Salmonella enterica Serovar Saintpaul as a Model
Source: mSphere. 2022 Jan 5;7(1):e00730-21. doi: 10.1128/msphere.00730-21 (PMC8731237; doi:10.1128/msphere.00730-21)
Supplement: TABLE S1 [file msphere.00730-21-st001.docx]

**Table S1.** Summary of the number of isolates and SNP clusters by phylogenetic groups and lineages.

| Phylogenetic groups/lineages | Number of isolates | | | Number of SNP clusters | | |
| --- | --- | --- | --- | --- | --- | --- |
|  | Human | Non-human | Total | HA | NHA | Total^a^ |
| **Group I** | 2797 | 1070 | 3867 | 24 | 21 | 178 |
| **-Lineage IA** | 1411 | 297 | 1708 | 11 | 9 | 80 |
| **-Lineage IB** | 806 | 602 | 1408 | 4 | 5 | 46 |
| **-Lineage IC** | 358 | 26 | 384 | 5 | 2 | 30 |
| **-Lineage ID** | 194 | 116 | 310 | 3 | 3 | 17 |
| **Group II** | 9 | 1 | 10 | 0 | 0 | 5 |
| **Group III** | 20 | 0 | 20 | 0 | 0 | 6 |
| **Group IV** | 569 | 245 | 814 | 5 | 2 | 22 |

^a^ The total number of SNP clusters does not equal the sum of the HA and NHA SNP clusters, as the majority of SNP clusters within a given phylogenetic group or lineage were not classified as HA nor NHA SNP clusters.
