# Supplementary figures and images for: Development of a Genomics-Based Approach To Identify Putative Hypervirulent Nontyphoidal Salmonella Isolates: Salmonella enterica Serovar Saintpaul as a Model
Source: mSphere. 2022 Jan 5;7(1):e00730-21. doi: 10.1128/msphere.00730-21 (PMC8731237; doi:10.1128/msphere.00730-21)

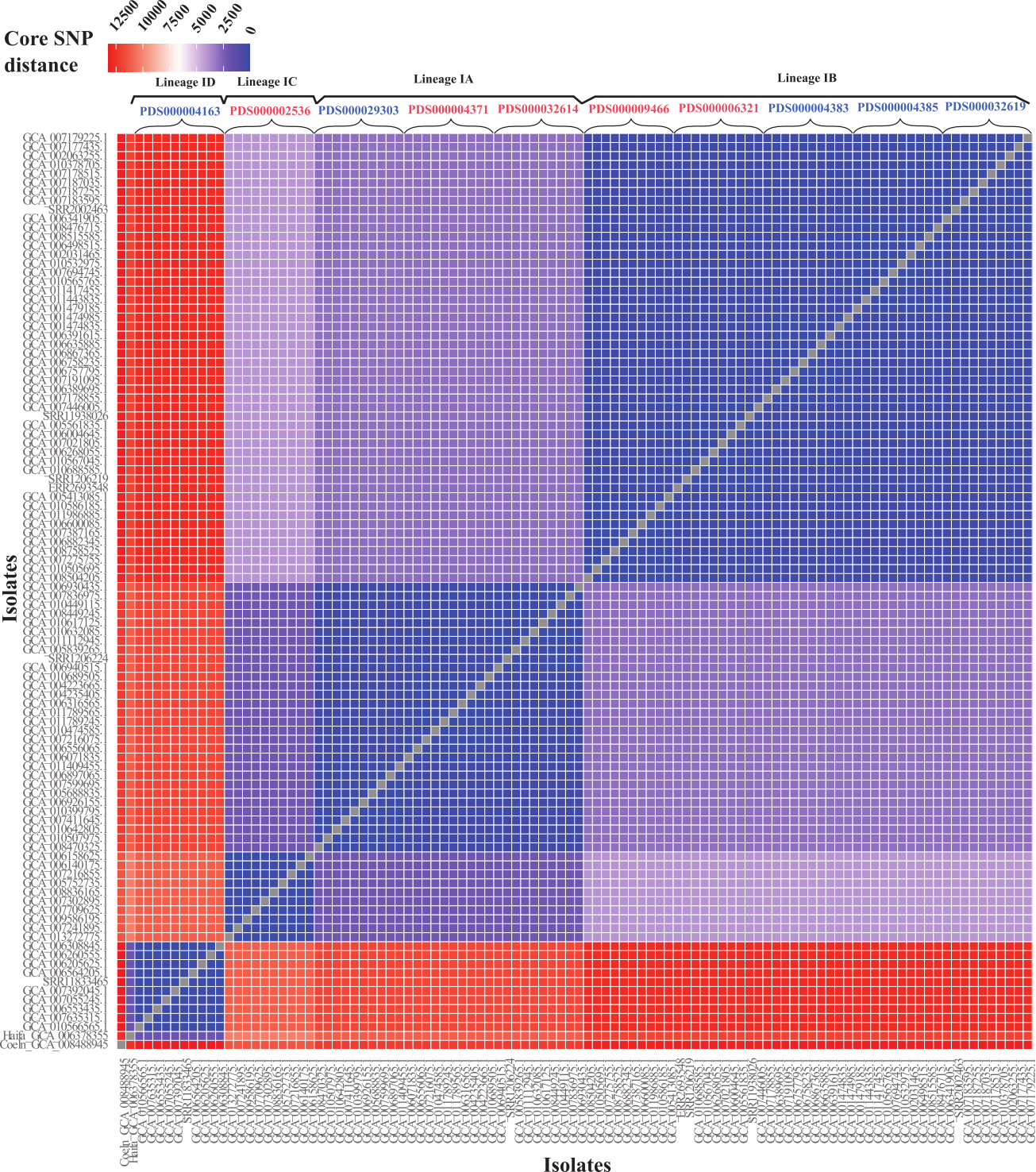

Supplement: FIG S1 [file msphere.00730-21-sf001.pdf]

**A**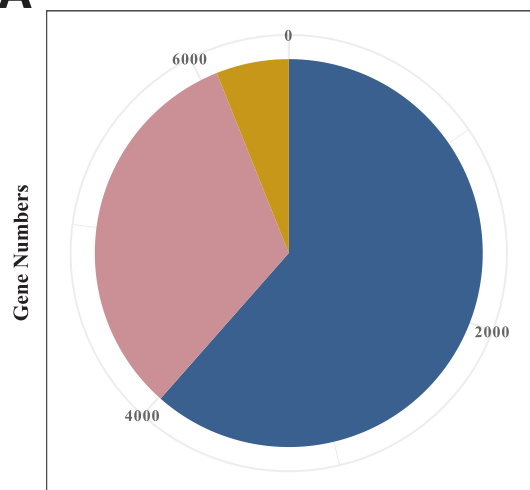

Core genes Shell genes Cloud genes

**B**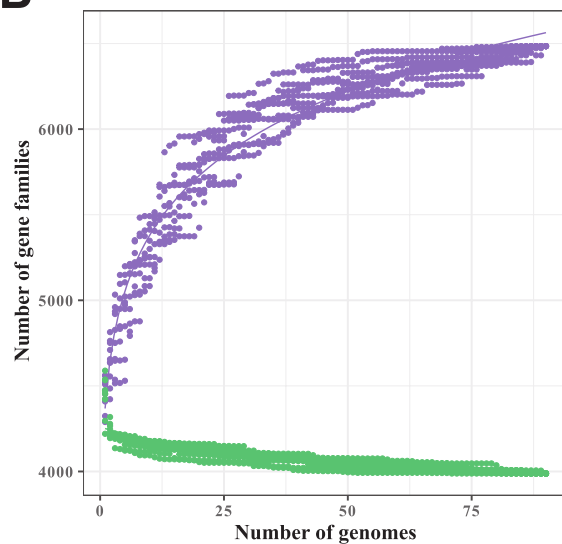

Core-genome Pan-genome

**C**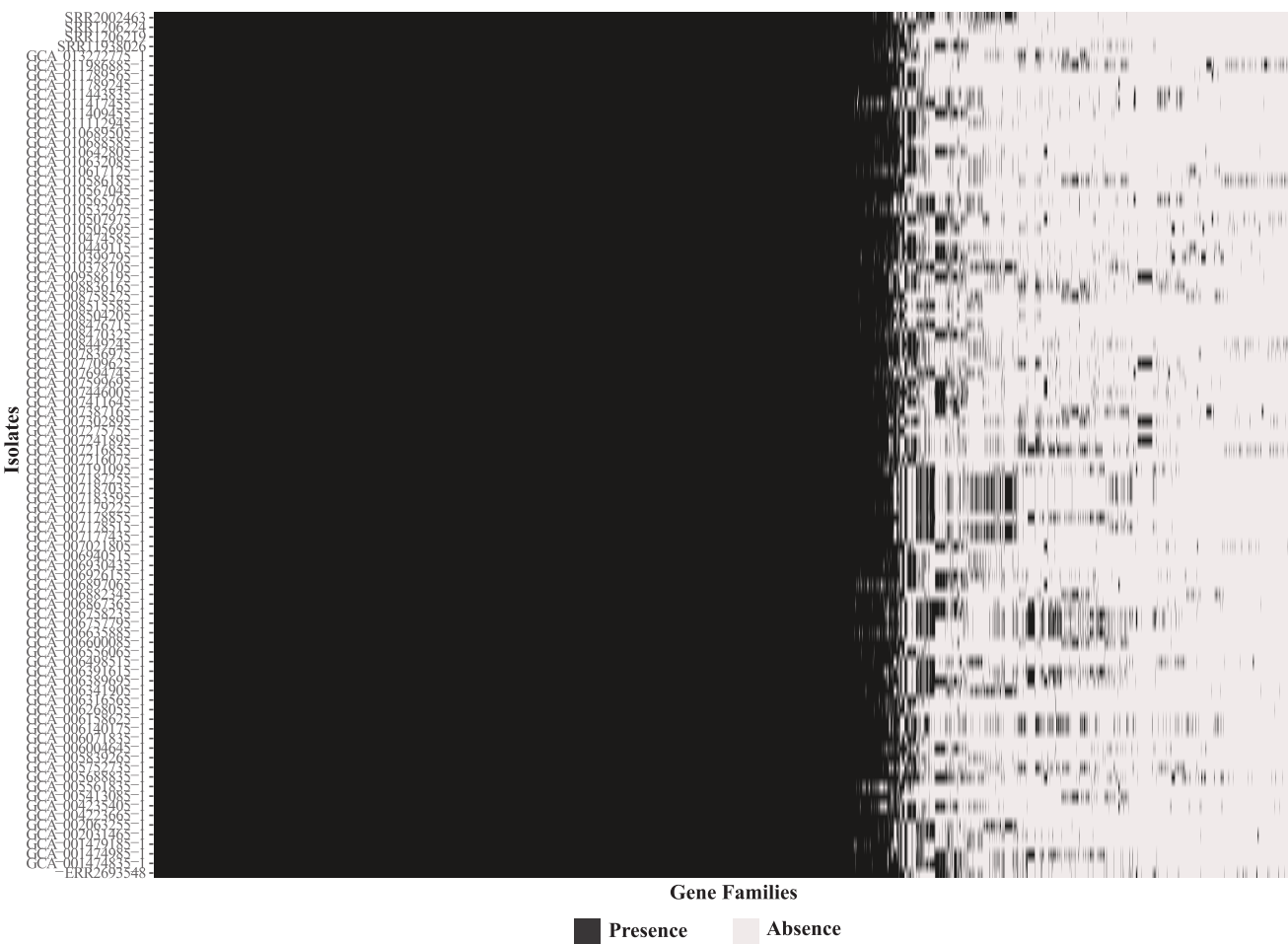

Supplement: FIG S2 [file msphere.00730-21-sf002.pdf]
