# Supplementary material for: Development of a Genomics-Based Approach To Identify Putative Hypervirulent Nontyphoidal Salmonella Isolates: Salmonella enterica Serovar Saintpaul as a Model
Source: mSphere. 2022 Jan 5;7(1):e00730-21. doi: 10.1128/msphere.00730-21 (PMC8731237; doi:10.1128/msphere.00730-21)
Supplement: TABLE S2 [file msphere.00730-21-st002.docx]

**Table S2.** The range and median number of HA/NHA-associated genes across the representative isolates of each SNP cluster.

| **SNP Cluster** | **Epi-type** | **Lineage** | **Genes overrepresented among HA isolates** | | | **Genes overrepresented among NHA isolates** | | |
| --- | --- | --- | --- | --- | --- | --- | --- | --- |
|  |  |  | Median no. (range) of total genes | Median no. (proportion of the total genes^a^) of plasmid-borne genes | Median no. (proportion of the total genes^a^) of prophage-borne genes | Median no. (range) of total genes | Median no. (proportion of the total genes^a^) of plasmid-borne genes | Median no. (proportion of the total genes^a^) of prophage-borne genes |
| PDS000002536 | HA | IC | 208 (104 - 222) | 0 (0%) | 93 (44.71%) | 32 (20 - 100) | 0 (0%) | 19.5 (60.94%) |
| PDS000004371 | HA | IA | 97 (95 - 128) | 0 (0%) | 43 (44.33%) | 11 (11 - 12) | 0 (0%) | 4 (36.36%) |
| PDS000032614 | HA | IA | 142 (138 - 162) | 0 (0%) | 72.5 (51.06%) | 12 (12 - 41) | 0 (0%) | 5 (41.67%) |
| PDS000006321 | HA | IB | 70 (66 - 77) | 0 (0%) | 46 (65.71%) | 30.5 (22 - 105) | 1.5 (4.92%) | 15 (49.18%) |
| PDS000009466 | HA | IB | 147 (77 - 169) | 0 (0%) | 53.5 (36.39%) | 19 (18 - 57) | 0 (0%) | 11 (57.89%) |
| PDS000029303 | NHA | IA | 97 (92 - 101) | 0 (0%) | 43 (44.56%) | 59 (52 - 123) | 21 (35.59%) | 4 (19.05%) |
| PDS000004383 | NHA | IB | 19 (17 - 25) | 0 (0%) | 0 (0%) | 265 (201- 342) | 47 (17.77%) | 113 (42.72%) |
| PDS000004385 | NHA | IB | 7 (6 - 8) | 0 (0%) | 0 (0%) | 174 (116 - 215) | 8 (4.60%) | 139 (79.89%) |
| PDS000032619 | NHA | IB | 7 (6 - 8) | 0 (0%) | 0 (0%) | 318 (225 - 339) | 18 (5.51%) | 162 (50.87%) |

^a^ The proportions are calculated using the median number of the total genes, plasmid-borne genes, and prophage-borne genes.
